# Tumor-associated macrophages promote chemoresistance to Paclitaxel via activating NOTCH2-JAG1 juxtacrine signaling

**DOI:** 10.1186/s12943-025-02546-w

**Published:** 2026-01-10

**Authors:** Fazhi Yu, Qin Zhou, Weiqiang Yu, Tong Zhou, Cheng Cao, Yijia Xie, Peng Zhang, Hanyuan Liu, Wei He, Aoxing Cheng, Xiaopeng Ma, Qingfa Wu, Qi Zhao, Jing Guo, Kaiguang Zhang, Ying Zhou, Jue Shi, Zhenye Yang

**Affiliations:** 1 Department of Digestive disease, Division of Life Sciences and Medicine, the First affiliated hospital of USTC, University of Science and Technology of China, Hefei, China; 2 State Key Laboratory of Immune Response and Immunotherapy, School of Basic Medical Sciences, Division of Life Sciences and Medicine, University of Science and Technology of China, Hefei, China; 3 Center for Reproduction and Genetics, Department of Obstetrics and Gynecology, Division of Life Sciences and Medicine, the First Affiliated Hospital of USTC, University of Science and Technology of China, Hefei, China; 4 Department of Obstetrics and Gynecology, Division of Life Sciences and Medicine, the First affiliated hospital of USTC, University of Science and Technology of China, Hefei, China; 5 Department of Epigenetics and Molecular Carcinogenesis, The University of Texas MD Anderson Cancer Center, Smithville, TX 78957 USA; 6 Department of General Surgery, the First affiliated hospital of University of Science and Technology of China, Anhui Provincial Hospital, Hefei, China; 7 MoE Frontiers Science Center for Precision Oncology, Faculty of Health Sciences, University of Macau, Taipa, Macau SAR China; 8 Center for Quantitative Systems Biology, Department of Physics, Hong Kong Baptist University, Hong Kong, China; 9 Institute of Cancer Research, Anhui Key Laboratory of Molecular Oncology, Division of Life Sciences and Medicine, University of Science and Technology of China, Hefei, 230027 PR China; 10 Center for Advanced Interdisciplinary Science and Biomedicine of IHM, Division of Life Sciences and Medicine, University of Science and Technology of China, Hefei, 230027 China

**Keywords:** Chemoresistance, Anti-mitotic drugs, Paclitaxel, Translational regulation, NOTCH2 signaling, Tumor-macrophage interaction

## Abstract

**Background:**

Taxane-based chemotherapy is a main treatment modality for ovarian cancer and other solid tumors, but chemoresistance limits the clinical efficacy. Studies have shown tumor interaction with macrophages in the tumor microenvironment (TME) plays a significant role in taxane resistance, yet the underlying molecular mechanisms are poorly understood.

**Methods:**

In this study, we employed translatome profiling of paclitaxel-treated cancer cells, live-cell imaging analysis, gene knockdown/knockout, and in vitro cancer-macrophage coculture assays to unravel a novel chemoresistance mechanism mediated by tumor-macrophage interaction via the NOTCH2-JAG1 axis. The in vitro data were further validated by multiple xenograft, syngeneic and patient-derived xenograft mouse tumor models of ovarian cancer as well as ovarian cancer patient samples.

**Results:**

We found paclitaxel selectively induced translational upregulation of NOTCH2 via cytoplasmic polyadenylation, and this NOTCH2 upregulation persisted after mitotic exit. Subsequent NOTCH2 activation by JAG1 expressed mainly on the neighboring macrophages promoted tumor cell survival and simulated cytokine release, such as CSF1 and IL-1β, that recruited JAG1-expressing macrophages, thus forming a positive feedback loop that further enhanced the pro-tumor NOTCH2 activity. Genetic depletion or pharmacological inhibition of NOTCH2 with the γ-secretase inhibitor attenuated macrophage infiltration and sensitized tumor response to paclitaxel in multiple preclinical models of ovarian cancer. Moreover, single-cell RNA sequencing analysis identified a JAG1-high macrophage subset that was enriched by paclitaxel treatment and attenuated by NOTCH inhibition. Clinically, high NOTCH2 expression in ovarian tumors was associated with recurrence and shorter progression-free survival of ovarian cancer patients.

**Conclusions:**

Paclitaxel-induced translational upregulation of NOTCH2 enables immediate juxtacrine activation by JAG1-positive macrophages, coupling tumor cell survival with immune remodeling in the tumor microenvironment to drive chemoresistance. Our results suggest NOTCH2 is a viable biomarker for paclitaxel resistance and that combining NOTCH2 inhibitor with taxane is an effective therapeutic strategy to selectively disrupt tumor-macrophage interaction and overcome macrophage-mediated taxane resistance in NOTCH2-positive tumors.

**Supplementary Information:**

The online version contains supplementary material available at 10.1186/s12943-025-02546-w.

## Introduction

Taxanes, including paclitaxel and its derivatives, are a class of anti-mitotic chemotherapeutics widely used as a major treatment modality for patients with solid tumors, including ovarian, breast, lung, and pancreatic cancers [1]. By binding to β-tubulin and disrupting microtubule dynamics, taxanes induce aberrant spindle formation and erroneous microtubule–kinetochore attachments, resulting in activation of the spindle assembly checkpoint (SAC) and prolonged mitotic arrest [2, 3]. Cells exposed to paclitaxel may die during mitosis, undergo abnormal multipolar division, or slip into an aberrant G1 state, where they may either die, arrest, or continue proliferate [4–10]. Despite their broad clinical application, the therapeutic efficacy of taxanes is frequently compromised by primary and acquired resistance, which remains a significant challenge for durable treatment responses.

Ovarian cancer represents one of the most lethal gynecological malignancies worldwide, with high-grade serous ovarian cancer (HGSOC) accounting for the majority of cases [11]. Most patients are diagnosed at advanced stages, when peritoneal dissemination and ascites are common. The current standard of care consists of cytoreductive surgery combined with platinum- and taxane-based chemotherapy, typically with paclitaxel as a first-line agent [12]. While initial responses are often favorable, relapse with chemoresistant disease is nearly universal, leading to poor long-term survival [11, 13]. Overcoming paclitaxel resistance in ovarian cancer, particularly in HGSOC, is therefore of paramount clinical importance.

The mechanisms underlying paclitaxel resistance have been extensively investigated. Tumor-intrinsic alterations such as increased drug efflux, activation of pro-survival pathways, evasion of apoptosis, altered tubulin expression or dynamics, and metabolic rewiring have all been implicated [14–17]. More recently, increasing attention has been directed toward the tumor microenvironment (TME) as a critical determinant of therapeutic response [18, 19]. For instance, stromal components, including cancer-associated fibroblasts and adipocytes, can secrete cytokines that attenuate drug-induced cytotoxicity. Moreover, immune cells in the TME, particularly tumor-associated macrophages (TAMs), have been shown to promote resistance to paclitaxel-induced growth arrest and cell death [20–24]. Several in vivo studies demonstrated that systemic depletion or repolarization of macrophages enhances tumor sensitivity to paclitaxel [20–24]. Nevertheless, the specific contact-dependent signaling mechanisms that mediate tumor-macrophage interactions and confer drug resistance remain poorly defined.

In this study, we sought to identify novel tumor-TME interactions that specifically contribute to paclitaxel resistance. Through translatome profiling of cancer cells under paclitaxel-induced mitotic arrest and functional co-culture analysis, we discovered that NOTCH2 is uniquely upregulated translationally during prolonged mitosis. Mechanistically, NOTCH2 on tumor cells engages its ligand JAG1, highly expressed on neighboring macrophages, to activate juxtacrine signaling in the post-mitotic G1 phase. This interaction promotes tumor cell survival after mitotic slippage and stimulates the secretion of cytokines that recruit and increase the immunosuppressive JAG1⁺ macrophages in the TME, thereby establishing a feed-forward loop that further enhances chemoresistance. Importantly, genetic knockdown or pharmacological inhibition of NOTCH2 with a pan-NOTCH inhibitor RO4929097 restored paclitaxel sensitivity and significantly suppressed tumor growth in multiple mouse models of ovarian cancer.

Collectively, our findings reveal a novel mechanism by which translational upregulation of NOTCH2 during mitotic arrest drives paclitaxel resistance through tumor-macrophage crosstalk in the TME. These results highlight NOTCH2 as both a biomarker for resistance and a promising therapeutic target, providing a rationale for precision combinatorial strategies that selectively disrupt NOTCH2-JAG1 signaling to overcome paclitaxel resistance and improve clinical outcomes in ovarian cancer.

## Results

### Paclitaxel induces translational upregulation of the transmembrane receptor NOTCH2 and is associated with chemoresistance in ovarian cancer patients

**Fig. 1:**
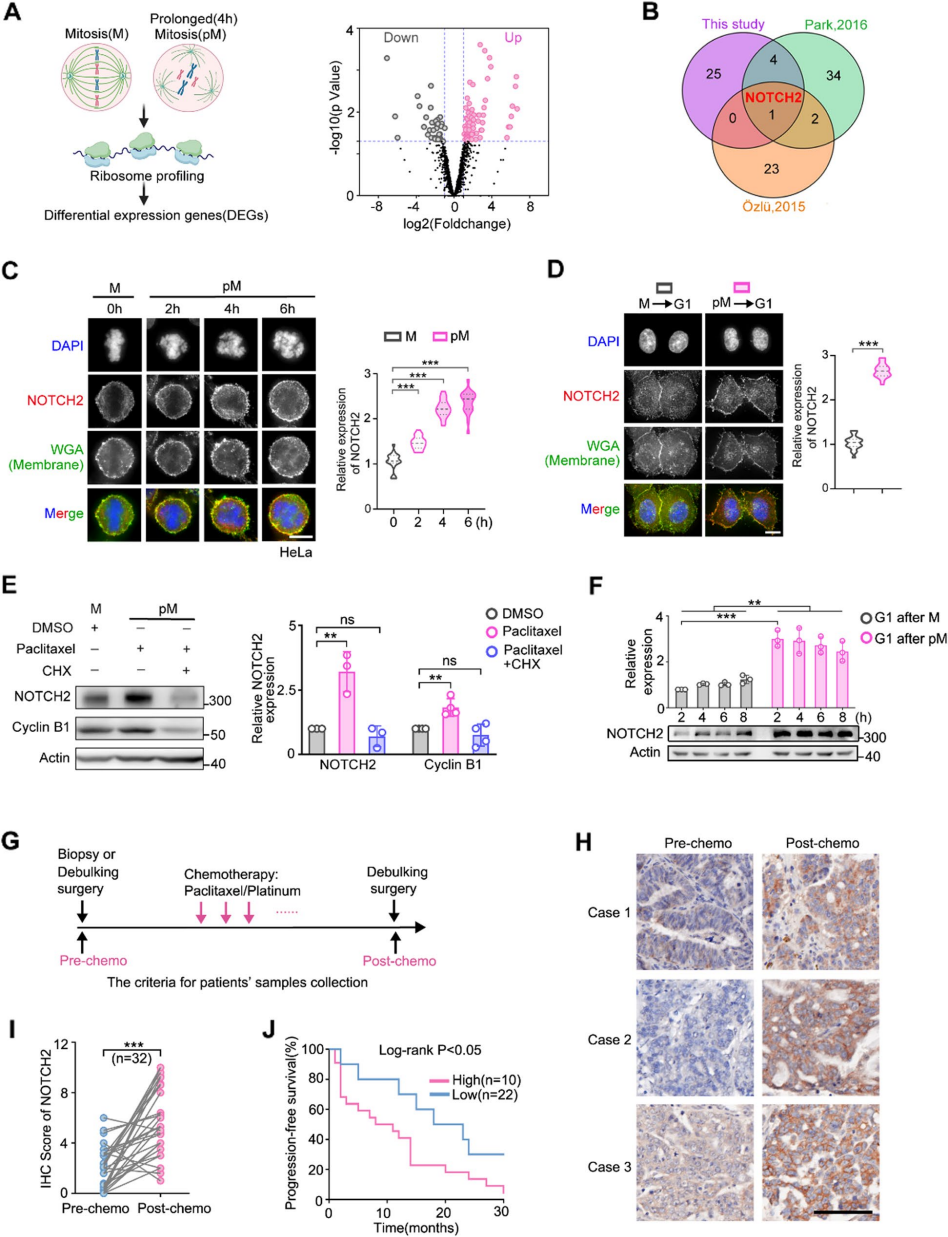
NOTCH2 translation is upregulated during paclitaxel-induced prolonged mitotic arrest and is associated with chemoresistance in ovarian cancer patients. **A** Ribosome profiling workflow and Volcano plot showing differentially expressed genes (DEGs) in prolonged mitotic (pM) versus mitotic (M) cells. Upregulated genes (log₂ fold change > 1.0, *p* < 0.05) and downregulated genes (log₂ fold change < − 1.0, *p* < 0.05) are highlighted. **B** Venn diagram showing the overlap of upregulated, membrane-related DEGs identified in this study with those reported in published datasets (Park et al., 2016 [25]; Özlü et al., 2015 [27]). **C** Immunofluorescence staining of NOTCH2 in interphase (G1) and paclitaxel-arrested mitotic HeLa cells, with cell membranes visualized using wheat germ agglutinin (WGA) staining. Representative images (left) and quantification (right) are shown. **D** Immunofluorescence staining of HeLa cells following mitotic exit induced by Reversine (500 nM). M→G1: normal mitotic exit into G1; pM→G1: exit from prolonged mitosis into an abnormal G1 state. Cell membranes are demarcated by WGA staining. Left: representative images; right: quantified NOTCH2 levels.**E** Western blot of NOTCH2 and Cyclin B1 in mitotic (M) and prolonged mitotic (pM) cells treated with DMSO or cycloheximide (CHX). Quantification is shown on the right. **F** Western blot analysis of NOTCH2 protein levels in HeLa cells treated with 0.5 µM nocodazole to induce mitotic arrest. Cells were harvested by shake-off and replated in fresh medium for the indicated times before collection. **G** Criteria for the collection of ovarian cancer patient samples. **H** Representative immunohistochemical staining of NOTCH2 in matched tumor specimens from ovarian cancer patients obtained before and after chemotherapy. Scale bars, 200 μm. Patients received standard adjuvant carboplatin–paclitaxel treatment. **I** Quantification of NOTCH2 IHC scores in paired tumor samples. **J** Kaplan-Meier survival analysis of ovarian cancer patients who had received the standard regimen of adjuvant carboplatin and paclitaxel. Patients were divided according to the IHC score of NOTCH2 (Optimal cut-off value was selected, detailed in Method) in their tumor tissues obtained before chemotherapy. Data in (C-F) are presented as mean ± SD from three independent experiments. Statistical significance was determined by two-tailed Student’s t-test (ns, not significant; *p < 0.05; **p < 0.01; ***p < 0.001) or log-rank test (J)

To identify cell surface receptor(s) that mediates cell-cell interaction in the TME and potentially contributes to paclitaxel resistance, we first conducted translatome profiling of upregulated membrane proteins/receptors in cancer cells during drug-induced prolonged mitotic arrest. We hypothesized that as prolonged mitotic arrest is the primary phenotype of paclitaxel, cellular alterations occurring during the mitotic arrest likely play crucial roles in engendering chemoresistance. And since transcription is largely silenced during mitosis, upregulation of pro-tumor molecular mediators probably occurs at the post-transcriptional level, such as translation. To perform the mitotic translatome analysis, mitotic cells were harvested by shake-off from untreated and paclitaxel-treated HeLa cells and subjected to ribosome profiling (Fig. 1A and Fig. S1A, B). More than nine thousands mRNAs were identified to be translated during normal mitosis, and 89% of these genes were also found in two previously published datasets, in which the translational landscape of mitosis was analyzed also by ribosome profiling [25, 26], validating that our data are well in line with results from previous studies (Fig. S1C). From the genes identified to be upregulated during prolonged mitosis (Fig. 1A and Table S1), cell membrane receptors were picked out and compared with those identified from two other available translatome analysis of HeLa cells in prolonged mitosis using ribosome profiling [25] and proteomics [27], respectively. The Venn diagram showed that NOTCH2 is the only upregulated membrane receptor identified by all three studies (Fig. 1B).

NOTCH2 is one of the four NOTCH signaling receptors that are frequently disregulated in cancers and involved in multiple oncogenic phenotypes, such as drug resistance via juxtacrine signaling at cell-cell contact [28–30]. We first validated NOTCH2 was upregulated at the protein level during mitotic arrest by performing immunofluorescence analyses in HeLa cells (Fig. 1C), and we also confirmed similar NOTCH2 upregulation in the ovarian cancer cell line, OVSAHO (Fig. S1D). And, we observed a progressive increase in NOTCH2 signal intensity in mitotic cells as mitotic arrest duration increased (Fig. 1C and Fig. S1D). To determine whether NOTCH2 upregulation persists after mitotic exit, we treated mitotic arrest cells with an MPS1 inhibitor to induce mitotic exit (Fig. 1D). Upon reattachment and re-entry into the G1 phase, NOTCH2 protein level remained elevated compared to that in the untreated interphase cells (Fig. 1D), illustrating sustained upregulation of NOTCH2 even after mitotic exit.

Moreover, similar to Cyclin B1, which is known to be actively translated during prolonged mitosis [16], the level of NOTCH2 protein (Fig. 1E), but not the mRNA (Fig. S1E), significantly increased during paclitaxel-induced prolonged mitotic arrest. The increase of NOTCH2 protein was abolished by cycloheximide treatment (Fig. 1E), indicating that NOTCH2 was upregulated at the protein synthesis level. While the protein level of NOTCH2 was already at a high level when cells entered the G1 phase (Fig. 1F), the mRNA of *NOTCH2* was not increased until six hours after mitotic exit (Fig. S1F), supporting that NOTCH2 was translationally upregulated during prolonged mitosis. We also confirmed the upregulation of NOTCH2 protein in the ovarian cancer cell lines, OVCAR8 and OVSAHO (Fig. S1G). In addition, we found NOTCH2 upregulation can be induced by other anti-mitotic compounds, such as nocodazole, STLC (Eg5 inhibitor) and BI2536 (PLK1 inhibitor) (Fig. S1H). Overall, our results showed that NOTCH2 is translationally upregulated during paclitaxel-induced prolonged mitotic arrest.

To evaluate the clinical relevance of NOTCH2, we examined its expression level in tumors from patients treated with taxanes. Paclitaxel-based chemotherapy is frequently used for advanced ovarian cancer, and we analyzed paired primary tissues and recurrent tissues from ovarian cancer patients by immunohistochemistry (IHC) staining (Fig. 1G). The IHC score (defined by expression levels detailed in Methods) revealed a significant correlation between NOTCH2 level and tumor recurrence in these patients after paclitaxel/platinum treatment (Fig. 1H and Fig. S1I). Few patients before the paclitaxel-based adjuvant therapy showed high NOTCH2 expression (Fig. 1H, I). When patients had tumor relapse after chemotherapy, the number of patients showing high NOTCH2 expression significantly increased, indicating that high NOTCH2 expression may be particularly associated with recurrent tumor after chemotherapy treatment (Fig. 1H, I). The upregulated NOTCH2 expression after chemotherapy is predominantly localized within the tumor cell regions, with minimal to no detectable staining in the stromal compartment or in macrophages, the major immune cells that infiltrated in the tumor region (Fig. S1J). The progression-free survival of patients with high NOTCH2 tumors before chemotherapy treatment was substantially shorter than patients with low NOTCH2 tumors (Fig. 1J), illustrating the correlation of NOTCH2 expression and patient overall prognosis. Together these clinical data indicate that NOTCH2 levels are significantly correlated with the tumor sensitivity to paclitaxel treatment for ovarian cancer patients. And NOTCH2 could be employed as a viable prognostic biomarker for predicting patient responses to taxane-based therapy.

### Polyadenylation-enhanced NOTCH2 translation during prolonged mitosis confers resistance to cell death in the subsequent interphase

**Fig. 2:**
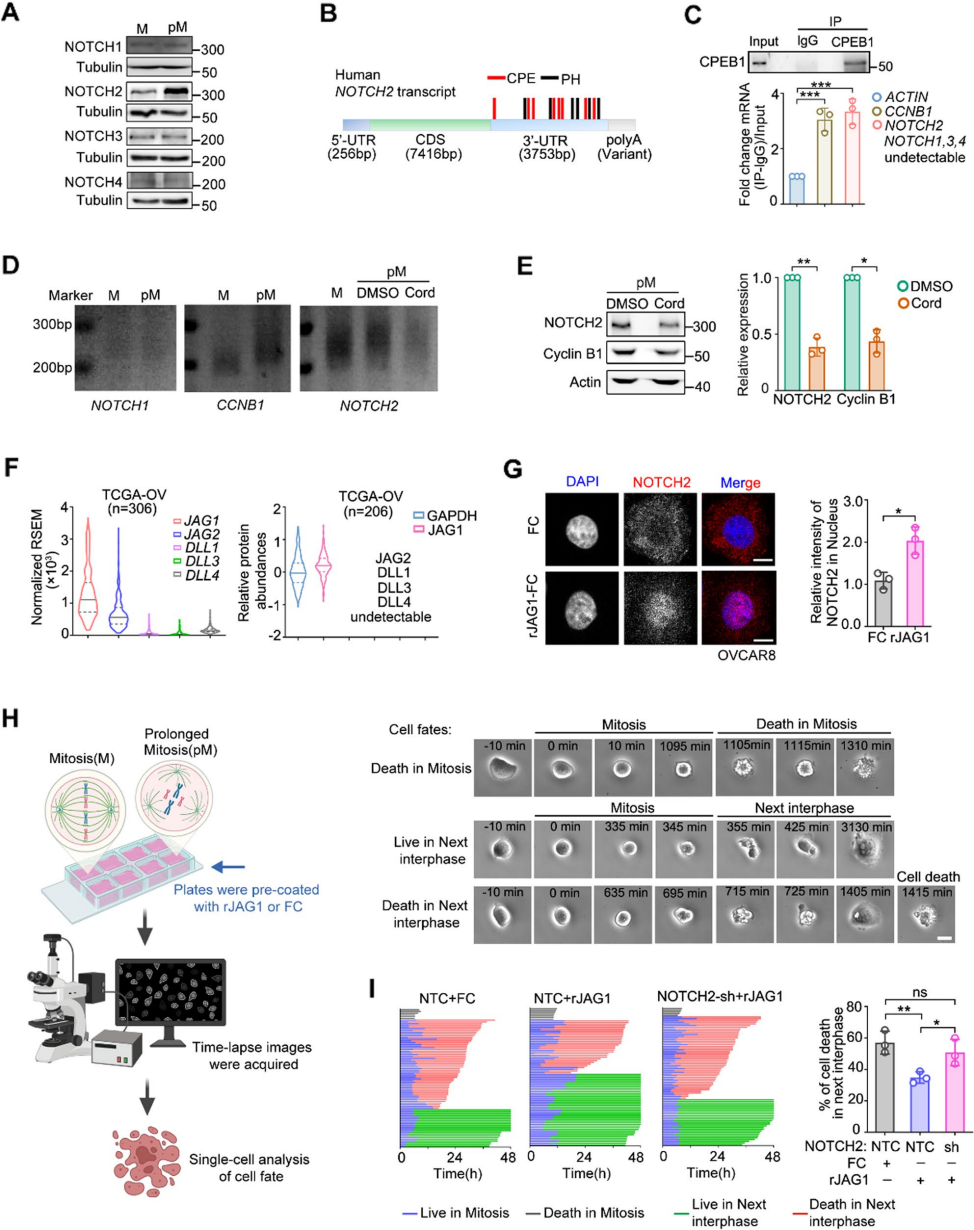
Polyadenylation-enhanced NOTCH2 translation during prolonged mitosis confers resistance to cell death in the subsequent interphase. **A** Western blot analysis of NOTCH receptor expression in HeLa cells during mitosis (M) and prolonged mitosis (pM). **B** Schematic representation of the number and position of CPE elements (sequence alignment: UUUUAU), polyadenylation hexanucleotide (PH, sequence alignment based on: AAUAAA) and polyadenylation signals in the 3’-UTR of the human NOTCH2 transcript. **C** RT-PCR detection of mRNA levels of the indicated genes after immunoprecipitation of CPEB1 in HeLa cells in prolonged mitosis. The CPEB1 IP efficiency was confirmed by western blot. **D** Poly(A) tail-length assay of *NOTCH1*, *CCNB1*, and *NOTCH2* transcripts in HeLa cells during mitosis (M) and prolonged mitosis (pM). Prolonged mitotic cells were treated with DMSO or 10 µM cordycepin (CPE inhibitor) for 4 h before collection. **E** Western blot analysis of NOTCH2 and Cyclin B1 in HeLa cells during prolonged mitosis treated with DMSO or cordycepin. Quantification is shown on the right. **F** Analysis of mRNA and protein levels of NOTCH ligands in ovarian cancer using TCGA data. **G** Immunofluorescence staining of NOTCH2 in OVCAR8 cells cultured on Fc or recombinant rJAG1-Fc for 12 h. Representative images (left) and quantification of nuclear NOTCH2 fluorescence intensity (right). Scale bar, 10 μm. **H** Schematic of experimental setup of cell fate tracking following mitotic arrest and rJAG1 stimulation. Cells were synchronized, and the mitotic cells were collected by shake-off before re-plating to 8-well plates or slides which were pre-coated with rJAG1. Live-cell imaging was used to track and analyze cells fates at the single cell level. Representative single cell images were showed. **I** Cell fate profiles of wild-type and NOTCH2-knockdown OVCAR8 cells treated with paclitaxel for 48 h in the presence of Fc or rJAG1-Fc, determined by live-cell imaging. Data are shown as mean ± SD from at least three independent replicates (C, D, E, G, I). p values were calculated using two-tailed Student’s t-tests (ns, not significant; *p < 0.05; **p < 0.01)

Uniquely among the four NOTCH receptors, NOTCH2 is the only one upregulated during prolonged mitosis (Fig. 2A), despite that both NOTCH2 and NOTCH3 are expressed in the ovarian cancer according to the TCGA dataset (Fig. S2A). To explore the origin of the differential translation of distinctive NOTCH receptors in prolonged mitosis, we examined the characteristics of *NOTCH* mRNA in comparison with the *CCNB1* mRNA, which encodes Cyclin B1, a highly translated protein in mitosis. *CCNB1* mRNA has multiple cytoplasmic polyadenylation element (CPE) and polyadenylation hexanucleotide (PH) elements. It has been reported that CPEB (cytoplasmic polyadenylation element binding protein) and CPSF (cleavage and polyadenylation specificity factor) promote the polyadenylation and translation of mRNA that contain sequences of CPE and PH [31]. Similar to *CCNB1*, there are eight CPE and five PH elements within the 3’ UTR of the *NOTCH2* mRNA (Fig. 2B and Fig. S2B). In contrast, few CPE or PH sequences were found in the mRNA of the other three *NOTCH* receptors (Fig. S2B). *CPEB* RNA Immunoprecipitation (RIP) assay demonstrated that similar to *CCNB1*, whose mRNA is known to bind CPEB, mRNA of *NOTCH2* also bound to CPEB1 in the mitotic cells (Fig. 2C), suggesting possible polyadenylation of *NOTCH2* mRNA during prolonged mitosis. We then analyzed the status of the polyA tail within the mRNA by PCR. Again, similar to *CCNB1*,* NOTCH2* mRNA showed higher polyadenylation during prolonged mitosis, whereas *NOTCH1* mRNA did not showed polyadenylation (Fig. 2D). In addition, CPEB inhibition with cordycepin, an inhibitor of CPEB-dependent polyadenylation, significantly attenuated NOTCH2 upregulation (Fig. 2E), illustrating that CPEB-directed polyadenylation promoted the synthesis of NOTCH2 during prolonged mitosis.

NOTCH and the downstream signaling cascades are activated by NOTCH binding to its cognate ligands expressed on the neighboring cells. Well-known NOTCH ligands include JAG1, JAG2, DLL1, DLL3 and DLL4 [29]. Based on analysis of the TCGA database of these ligands, we found JAG1 exhibited high expression in ovarian cancer at both the mRNA and protein level (Fig. 2F). To investigate the role of NOTCH2 activation in the paclitaxel response, we coated coverslips with recombinant rJAG1 and used them to stimulate NOTCH2 signaling in the ovarian cancer cell lines, OVCRA8 and OVSAHO, as well as HeLa cells. This treatment led to nuclear accumulation of NOTCH2, a signature of NOTCH signaling activation, in all three cell lines, confirming pathway activation upon interaction with rJAG1 (Fig. 2G and Fig. S2C–D).

To assess whether NOTCH2 activation affects paclitaxel-induced mitotic arrest and survival, we first synchronized ovarian cancer cells (OVCAR8 and OVSAHO) and treated them with paclitaxel to induce mitotic arrest. Mitotically arrested cells were then harvested by shake-off and seeded onto rJAG1-coated dishes. Live-cell imaging analysis was subsequently performed to monitor cell cycle progress and post-mitotic cell fates (Fig. 2H). Note that in this experimental setup, cells first underwent paclitaxel-induced mitotic arrest before encountering the rJAG1 stimulation. We found that cells plated onto the rJAG1-coated dishes, but not the Fc-coated control, exhibited significantly reduced cell death in the post-mitotic G1 phase under paclitaxel treatment (Fig. 2I and Fig. S2C, D). And this pro-survival effect was abolished in the NOTCH2-knockdown cells, indicating that it is specifically mediated by NOTCH2 signaling (Fig. 2I and Fig. S2C, D). We also confirmed that NOTCH2 knockdown did not affect the baseline cell cycle progression, as wild-type and NOTCH2-deficient (NOTCH2-knockdown) cells displayed similar cell cycle profiles in the absence of paclitaxel (Fig. S2E, F). Therefore, the pro-survival effect of NOTCH2 signaling is induced and specific to paclitaxel treatment.

### JAG1 expressed on the neighboring macrophages is primarily responsible for activating NOTCH2 and promoting paclitaxel resistance in vivo

**Fig. 3:**
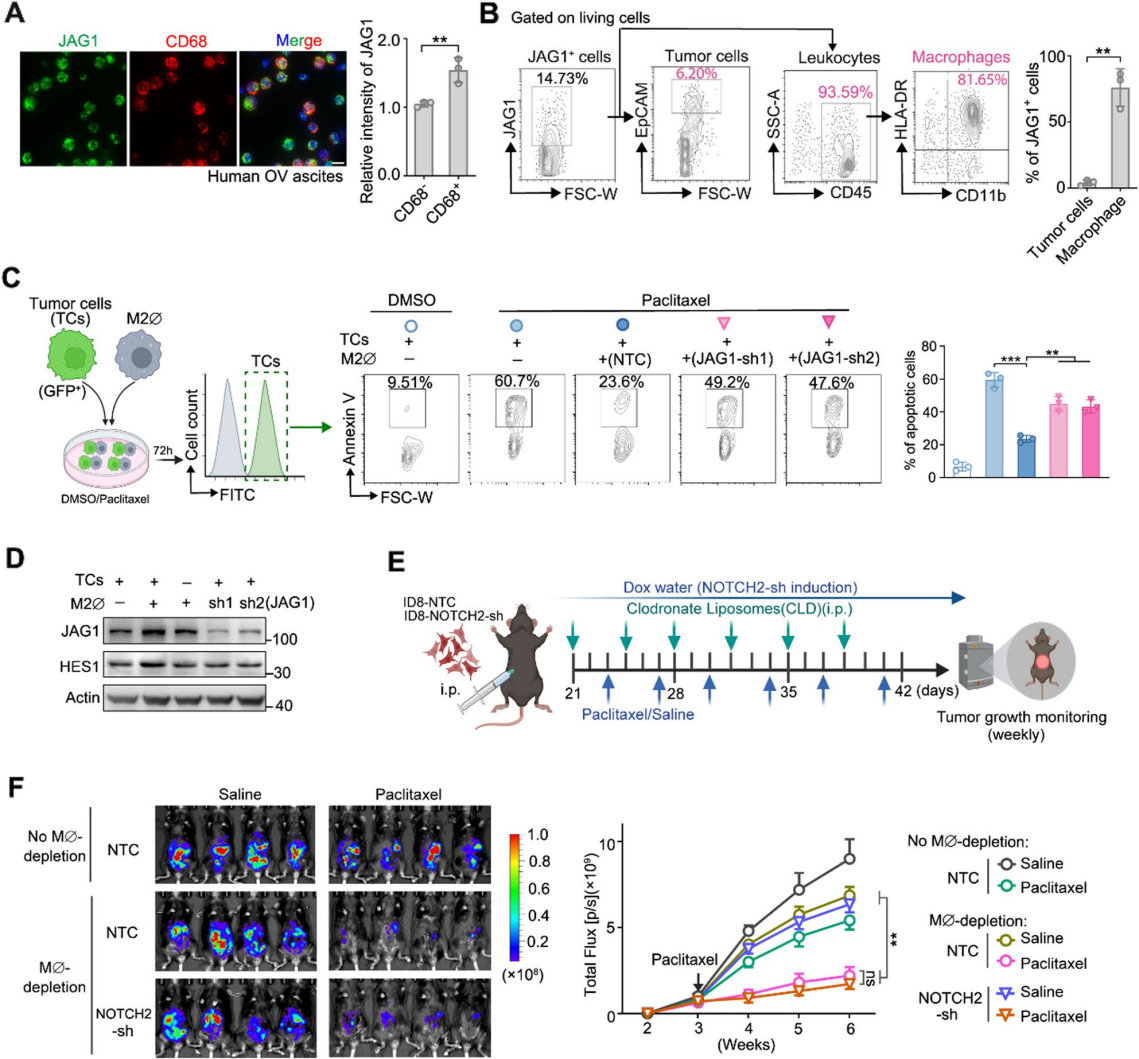
JAG1 expressed on the macrophages is primarily responsible for activating NOTCH2 signaling and promoting paclitaxel resistance in vivo. **A** Immunofluorescence staining of JAG1 in cells from ascites of patients with stage III/IV high-grade serous ovarian carcinoma before paclitaxel-based chemotherapy. Representative images and quantification of JAG1 intensity are shown (three ascites per group). Scale bar, 10 μm. **B** Flow cytometry analysis of the percentage of tumor cells and macrophages among the JAG1^+^ cells from ascites of patients with stage III/IV high-grade serous ovarian carcinoma before paclitaxel-based chemotherapy. **C** Co-culture assay of GFP-positive OVCAR8 cells with THP-1-derived M2-like-macrophage. OVCAR8-GFP cell death was detected and quantified using flow cytometry analysis of Annexin V staining. **D** Western blot analysis of JAG1, HES1 and Actin (loading control) for OVCAR8 cells in co-culture with THP-1-derived M2-like macrophages. **E** Schematic of the ID8 ovarian tumor model. Macrophages were depleted with clodronate liposomes. **F** Representative bioluminescence images of mice bearing ID8-luciferase tumors with or without macrophage depletion (left). Tumor growth curves are shown on the right (*n* = 6 per group, mean ± SEM). Data in (B-D) are shown as mean ± SD from at least three independent experiments. p values were calculated by two-tailed Student’s t-test (ns, not significant; **p < 0.01; ***p < 0.001)

By performing immunostaining of ascites from ovarian cancer patients (Fig. 3A), we found JAG1 was even more highly expressed in CD68^+^ macrophages in the TME (Fig. 3A). This observation was further confirmed by flow cytometry analysis, which showed a significantly higher level of JAG1 expressed on macrophages than that on the tumor cells from the ascites of human ovarian tumors before treatment with paclitaxel (Fig. 3B). These data suggested JAG1 expressed on the neighboring macrophages is potentially the major activator of in vivo NOTCH2 signaling in the TME that underlies paclitaxel resistance.

To confirm and explore the role of JAG1-expressing macrophages in the observed paclitaxel resistance, we first used in vitro co-culture models of OVCAR8 cells with M2-like macrophages (M2∅) derived from THP-1 (a human leukemic monocyte cell line, Fig. S3A). Tumor cell death was quantified by flow cytometry using Annexin V staining of the OVCAR8 cells (Fig. 3C, the OVCAR8 cells were labelled by GFP to distinguish them from the macrophages). We found that M2-like macrophages had a moderately higher JAG1 expression than the cancer cells (Fig. 3D). While the presence of M2-like THP-1-derived macrophages significantly attenuated OVCAR8 cell death, knocking down JAG1 in the THP-1-derived macrophages significantly increased cell death and attenuated the survival of OVCAR8 cancer cells in response to paclitaxel (Fig. 3C). The pro-survival effect exerted by the M2-like macrophages was also abrogated when NOTCH2 was knocked down in OVCAR8 (Fig. S3B, C). These findings were further validated using human PBMCs-derived primary M2-like macrophages in co-culture with the OVCAR8 cells (Fig. S3D, E), pointing to JAG1 expressed on the M2-like macrophages being an important activator of NOTCH2 signaling.

To further examine the macrophage involvement in mediating paclitaxel resistance in vivo, we depleted macrophages in ID8 syngeneic mouse model of ovarian cancer using liposomes-clodronate (Fig. 3F and Fig. S3F). Similar to the ovarian cancer patient samples discussed in Fig. 3A-B, analysis of the ascites of the ID8 mouse tumor model showed JAG1 was mainly expressed on the macrophages (Fig. S3G, H). In the absence of macrophages, the in vivo tumors were substantially more sensitive to paclitaxel-induced growth arrest, and knocking down NOTCH2 in the tumor cells did not further enhance the tumor sensitivity to paclitaxel (Fig. 3F). Overall, our data, in particular from the in vivo tumor model, suggest that tumor-macrophage interaction is the primary activator of NOTCH2-JAG1 juxtacrine signaling in the TME that mediates paclitaxel resistance.

### NOTCH2 signaling activates pro-survival pathways and stimulates the recruitment of pro-tumor macrophages

**Fig. 4:**
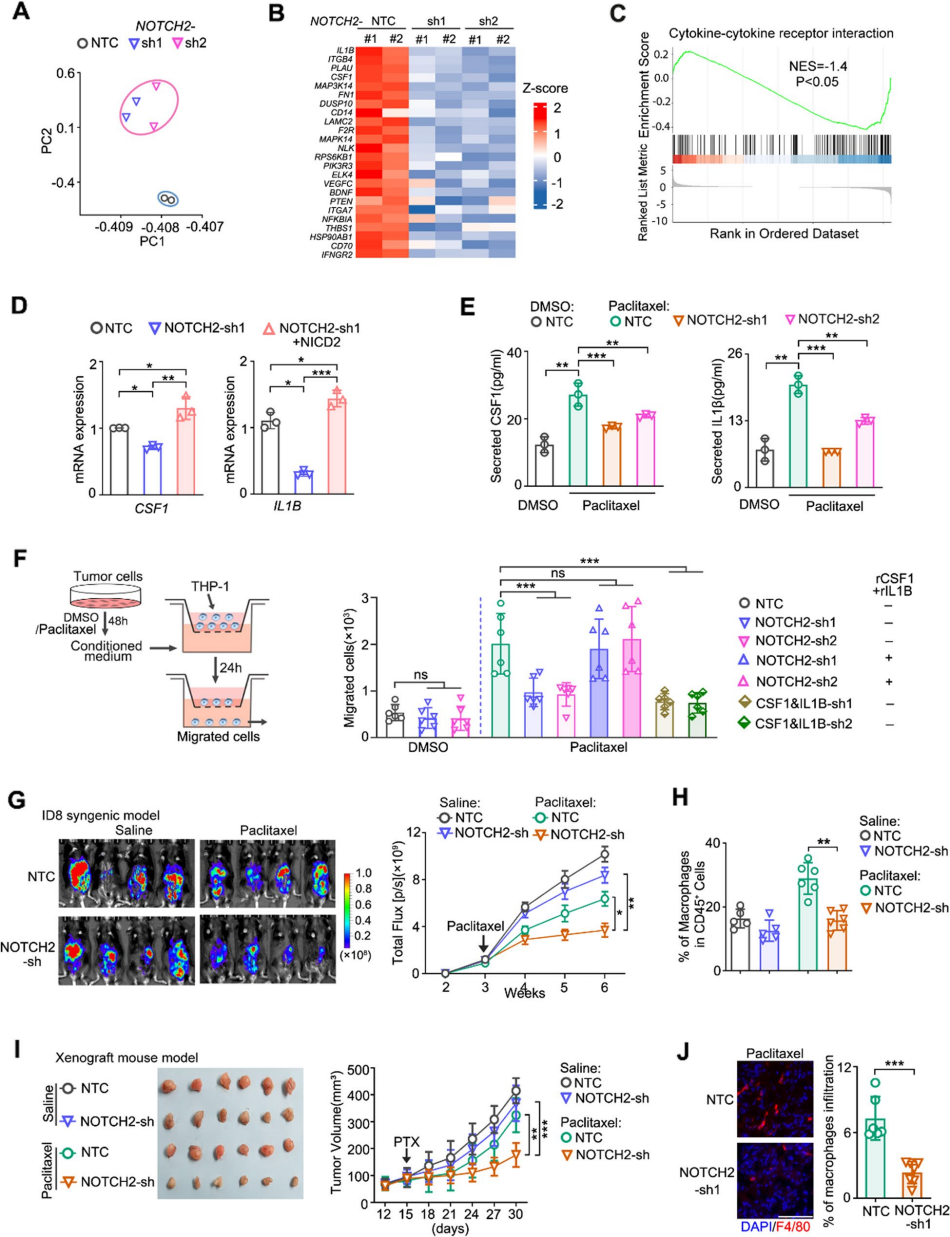
NOTCH2 upregulation activates pro-survival pathways and stimulates the recruitment of pro-tumor macrophages. **A** Principal component analysis (PCA) of RNA-seq data from wild-type and NOTCH2-knockdown OVCAR8 cells using two independent shRNA clones. **B** Heatmap illustrates differentially expressed genes (DEGs) in OVCAR8 cells transfected with NOTCH2-shRNA or non-target control (NTC) vector. **C** Gene set enrichment analysis (GSEA) of RNA-seq data from OVCAR8 cells with or without NOTCH2 knockdown. **D** RT-PCR analysis of *CSF1* and *IL1B* mRNA in OVCAR8 cells transfected with vector or NICD2-expression construct, treated with 10 nM paclitaxel for 48 h. **E** ELISA quantification of secreted CSF1 and IL1β in culture medium from OVCAR8 cells with or without NOTCH2 knockdown, treated with DMSO or paclitaxel for 48 h. **F** Transwell chemotaxis assay measuring THP-1 migration in response to conditioned medium from OVCAR8 cells treated with vehicle or paclitaxel. Experimental scheme (left) and quantification (right) are shown. **G** Bioluminescence images of ID8-luciferase tumors with inducible NOTCH2 knockdown or control (left) and corresponding tumor growth curves (right) (*n* = 5, mean ± SEM). **H** Quantification of macrophages in ascites from ID8 tumor-bearing mice in (G). **I** Representative dissected tumors (left), tumor growth curves (middle) from OVCAR8 xenograft mice (*n* = 6, mean ± SD). Mice carrying inducible NOTCH2-knockdown or control cells received paclitaxel (10 mg/kg, i.p., every 2 days for 2 weeks) after tumors reached ~ 100 mm³. **J **Immunofluorescence staining of tumor sections from OVCAR8 xenograft mice using F4/80 (macrophages) and DAPI, with quantification of F4/80⁺ cells (right). Scale bar, 20 μm. Data are shown as mean ± SD. Data in (D-F) are shown as mean ± SD from at least three independent experiments. p values were calculated by two-tailed Student’s t-test (ns, not significant; *p < 0.05; **p < 0.01; ***p < 0.001)

To pinpoint how activation of NOTCH2 signaling confers paclitaxel resistance at the molecular and cellular level, we investigated the downstream signaling pathways of NOTCH2 using transcriptome analysis of NOTCH2-depleted cells. Principal component analysis of the transcriptome data from NOTCH2-knockdown OVCAR8 cells using two different NOTCH2 shRNA constructs gave consistent results (Fig. 4A, Fig. S4A). In addition to downregulation of the pro-survival PI3K-AKT signaling and MAPK signaling in the NOTCH2-knockdown cells (Fig. S4B), we found considerable downregulation of cytokines, such as *CSF1* and *IL1B* , upon NOTCH2 knockdown (Fig. 4B). Further GSEA pathway analysis revealed that cytokine-cytokine receptor interaction pathway was indeed significantly downregulated under NOTCH2 knockdown (Fig. 4C). We subsequently confirmed, by qPCR and ELISA, that paclitaxel treatment induced expression of CSF1 and IL-1β, and NOTCH2 depletion markedly reduced their expression in OVCAR8 cells (Fig. 4D, E and Fig. S4C). Moreover, overexpression of the active intracellular domain of NOTCH2, NICD2, rescued the expression of *CSF1 and IL1B* under NOTCH2 depletion (Fig. 4D and Fig. S4D). Analysis of the TCGA dataset also showed strong correlation of NOTCH2 level with *CSF1/ IL1B* levels (Fig. S4E) in ovarian cancer.

CSF1 and IL-1β are known to recruit pro-tumor M2-like macrophages [20, 32], implicating a possible immunomodulatory role of NOTCH2 activation to increase pro-tumor macrophages and augment the pro-survival tumor-macrophage interactions in the TME. To characterize the effect of NOTCH2 activation in recruiting macrophages, conditioned medium from control and NOTCH2-depleted OVCAR8 cells were collected and their effects on the migration of THP-1 were measured by a transwell assay (Fig. 4F). Compared to medium from the paclitaxel-treated wild-type cells, medium from the NOTCH2-depleted cells under paclitaxel treatment showed significantly less effect in promoting THP-1 migration (Fig. 4F). There was no such difference for medium from DMSO-treated wild-type and NOTCH2-depleted cells (Fig. 4F). Moreover, addition of recombinant CSF1 and IL-1β to the medium of NOTCH2-depleted cells resulted in an extent of THP-1 migration similar to that induced by the wild-type medium (Fig. 4F). And concurrent depletion of CSF1 and IL-1β in cancer cells significantly reduced the migration of THP-1 cells (Fig. 4F and S4F). Together these data illustrate that NOTCH2 signaling stimulates macrophage recruitment via upregulating cytokines, such as CSF1 and IL-1β.

We next examined the potential role of NOTCH2 activation in promoting macrophage recruitment in the ID8 syngeneic mouse model of ovarian cancer under paclitaxel treatment. Depletion of NOTCH2 by shRNA did not affect tumor growth in the control mice, but significantly attenuated the tumor growth in the paclitaxel-treated mice (Fig. 4G and S4G), again confirming that NOTCH2 is involved in promoting paclitaxel resistance in vivo. We also found that in the ID8 mouse ascites, only the number of macrophages, but not the other immune cell types, was significantly reduced in mice bearing the NOTCH2-depleted tumor cells upon paclitaxel treatment (Fig. 4H and Fig. S4H). Similar results were also observed for the ID8 bulk tumor samples. Among the intra-tumoral immune cells, the number of macrophages exhibited the most significant increase upon paclitaxel treatment and this increase was substantially attenuated in the NOTCH2-knockdown tumors (Fig. S4I, J).

In addition to the ID8 mouse tumor model, we also examined the involvement of macrophage recruitment in mouse xenograft tumor models derived from OVCAR8 cells. The results showed that NOTCH2 knockdown significantly attenuated tumor growth under paclitaxel treatment in the xenograft tumor models (Fig. 4I and S4K). And knocking down NOTCH2 significantly reduced the number of tumor-infiltrated macrophages under paclitaxel treatment (Fig. 4J), again confirming the in vivo role of NOTCH2 signaling in promoting macrophage recruitment to the TME. Overall, both the in vitro and in vivo data showed that paclitaxel resistance is mediated and enhanced by NOTCH2-activated macrophage recruitment to the TME.

### NOTCH2 inhibitor sensitizes tumor response to Paclitaxel in multiple mouse tumor models of ovarian cancer

**Fig. 5:**
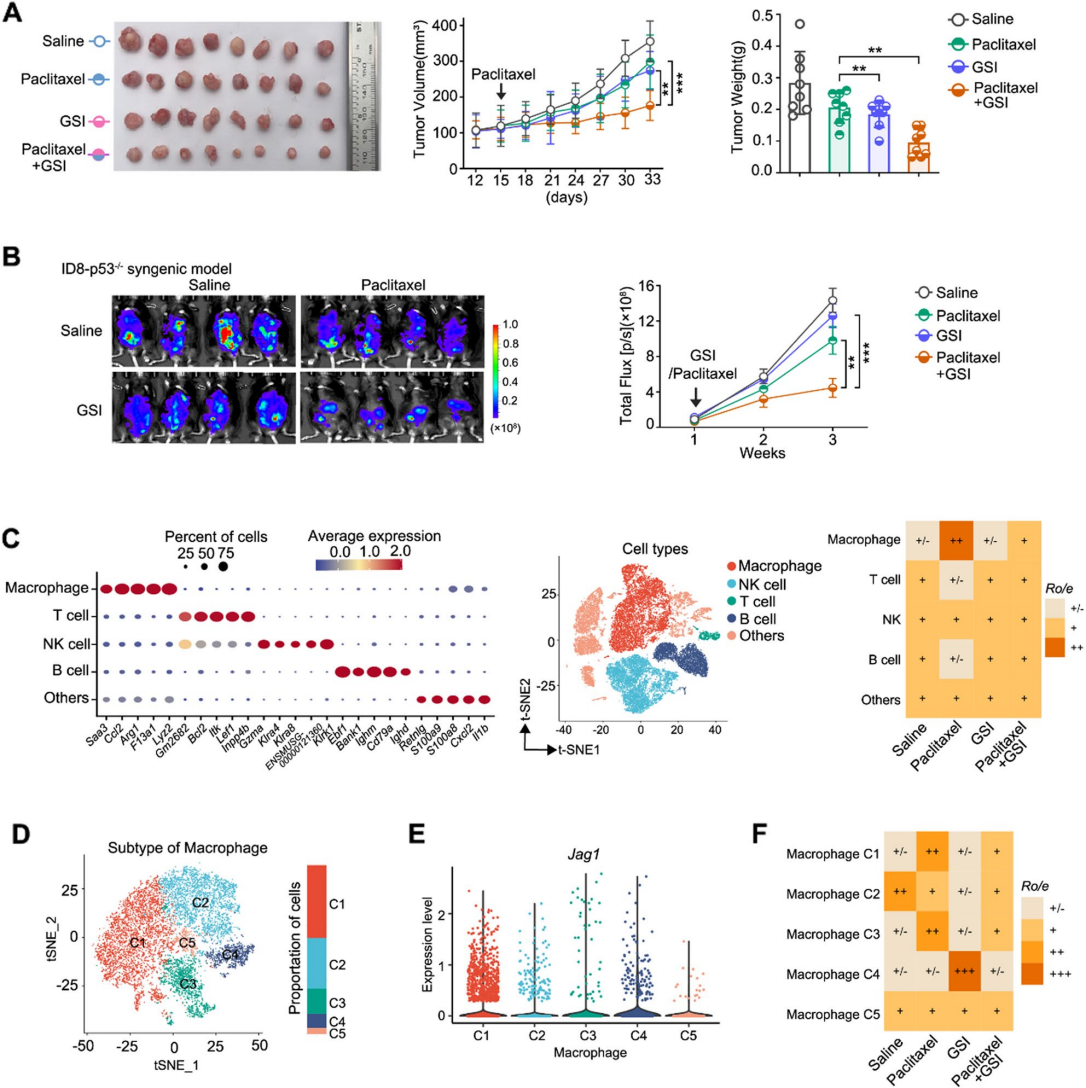
NOTCH2 inhibitor sensitizes tumor response to paclitaxel in xenograft and syngeneic mouse tumor models of ovarian cancer.** A** Representative tumors (left), tumor growth curves (middle), and tumor weights (right) from OVCAR8 xenograft-bearing mice treated with paclitaxel, GSI, or the combination (*n* = 8 per group). Growth curves are shown as mean ± SEM; tumor weights as mean ± SD. **B** Bioluminescence images of ID8-p53⁻/⁻-luciferase tumors (left) and corresponding tumor growth curves (right) after two weeks of treatment (*n* = 6 per group, mean ± SEM). **C** Immune landscape of ascites from the ID8-p53⁻/⁻ model under different treatments. Left: dot plot of canonical marker genes used for cell type annotation (dot size, proportion of cells; color, average expression). Middle: UMAP clustering of immune cell populations. Right: relative enrichment (Ro/e) of immune cell types across treatment groups. **D** UMAP plots showing five macrophage subsets identified in ascites from the ID8-p53⁻/⁻ model. **E** Violin plots of *Jag1* expression across the five macrophage subsets. **F** Relative enrichment (Ro/e) of macrophage subsets (C1–C5) under different treatments. Ro/e represents the ratio of observed to expected frequency (values > 1, over-representation; values < 1, under-representation). Heatmap scale: grey (+/–, no change), light orange (+), orange (++), dark orange (+++). p values were calculated by two-tailed Student’s t-test (**p < 0.01; ***p < 0.001)

As there is no NOTCH2-specific small-molecule inhibitor currently available, we used a pan-NOTCH inhibitor, RO4929097 (a Gamma-secretase inhibitor (GSI)), as a proof-of-concept, to evaluate the combinatorial efficacy of NOTCH2 inhibition and paclitaxel. NOTCH2 inhibition by GSI treatment was confirmed by the reduced expression of the NOTCH2 target gene, HES1 (Fig. S5A). In the mouse xenograft tumor model of OVCAR8, combinatorial treatment of GSI plus paclitaxel significantly enhanced the tumor growth inhibition, as compared to paclitaxel or GSI treatment alone (Fig. 5A). This synergistic inhibitory effect was not further enhanced when NOTCH2 was depleted, indicating that NOTCH2 is primarily responsible for the pan-NOTCH inhibition effect of GSI (Fig. S5B). We also evaluated the combinatorial treatment effect in the ID8 syngeneic mouse tumor model (Fig. S5C). Again, compared to paclitaxel treatment alone, GSI plus paclitaxel showed significantly higher attenuation of tumor growth (Fig. S5C) as well as a significant reduction of macrophages in the paclitaxel-treated tumor (Fig. S5D), illustrating that blocking NOTCH2 signaling damped the recruitment of pro-tumor macrophages under paclitaxel treatment.

To better recapitulate the loss-of-function of *TP53* commonly observed in high-grade serous ovarian cancer (HGSOC) and evaluate whether p53 functional status impacts the NOTCH2-JAG1 signaling axis that mediates paclitaxel resistance, we established a syngeneic mouse tumor model using ID8 cells (p53 wildtype) with stable *Trp53* knockout (ID8-p53^⁻/⁻^) [33] to evaluate the combinatorial efficacy of GSI and paclitaxel. The results showed evident synergistic effect of GSI and paclitaxel in attenuating tumor growth in the ID8-p53^⁻/⁻^ tumor model, similar to that observed in the p53-wildtype ID8 tumor model (Fig. 5B), indicating that the combined treatment of GSI and paclitaxel may be broadly effective in NOTCH2-positive ovarian cancer, irrespective of the p53 functional status.

In addition, we performed single-cell RNA sequencing (scRNA-seq) analysis of the ID8-p53⁻/⁻ mouse ascites to examine the tumor immune microenvironment under the different treatment conditions (e.g., paclitaxel, GSI, and paclitaxel + GSI) (Fig. 5C). Clustering analysis of the scRNAseq results enabled the identification of different immune cell types and their respective abundance under the different treatment conditions (Fig. 5C). Moreover, based on the expression levels of a panel of macrophage markers, we further classified the macrophages into five distinctive macrophage subsets, denoted as C1, C2, C3, C4 and C5 (Fig. 5D and S5E), among which the C1 subset exhibited the highest expression of *Jag1* (Fig. 5E). By comparing the transcriptional profiles of *Jag1⁺* and *Jag1*⁻ macrophages, we found that *Jag1*⁺ macrophages exhibited a distinct pro-tumorigenic gene signature, characterized by the upregulation of genes involved in oxidative stress response (e.g., *Hmox1*,* Gclm*,* Fth1*), lipid metabolism (*Fabp5*,* Cd36*,* Lpl*), extracellular matrix remodeling (*Fmnl2*,* Adam8*), and inflammation modulation (*Ccl8*,* Saa3*,* Marco*) (Fig. S5F). Conversely, *Jag1*⁻ macrophages preferentially expressed immuno-regulatory and chemotactic genes, including *Ccr2*,* Plac8*,* Vcan*,* Sirpd*, and *Junb* (Fig. S5F). These transcriptional differences highlight that *Jag1* expression delineates a functionally specialized macrophage subset that promotes tumor progression and therapeutic resistance. Notably, the *Jag1*^+^ C1 subset was significantly enriched upon paclitaxel treatment, and this increase was attenuated by combinatorial treatment with GSI (Fig. 5F), suggesting that the combinatorial treatment of GSI plus paclitaxel suppresses the activity of these pro-tumor macrophages.

### NOTCH2 inhibitor sensitizes ovarian cancer patient-derived xenograft tumor response to Paclitaxel

**Fig. 6:**
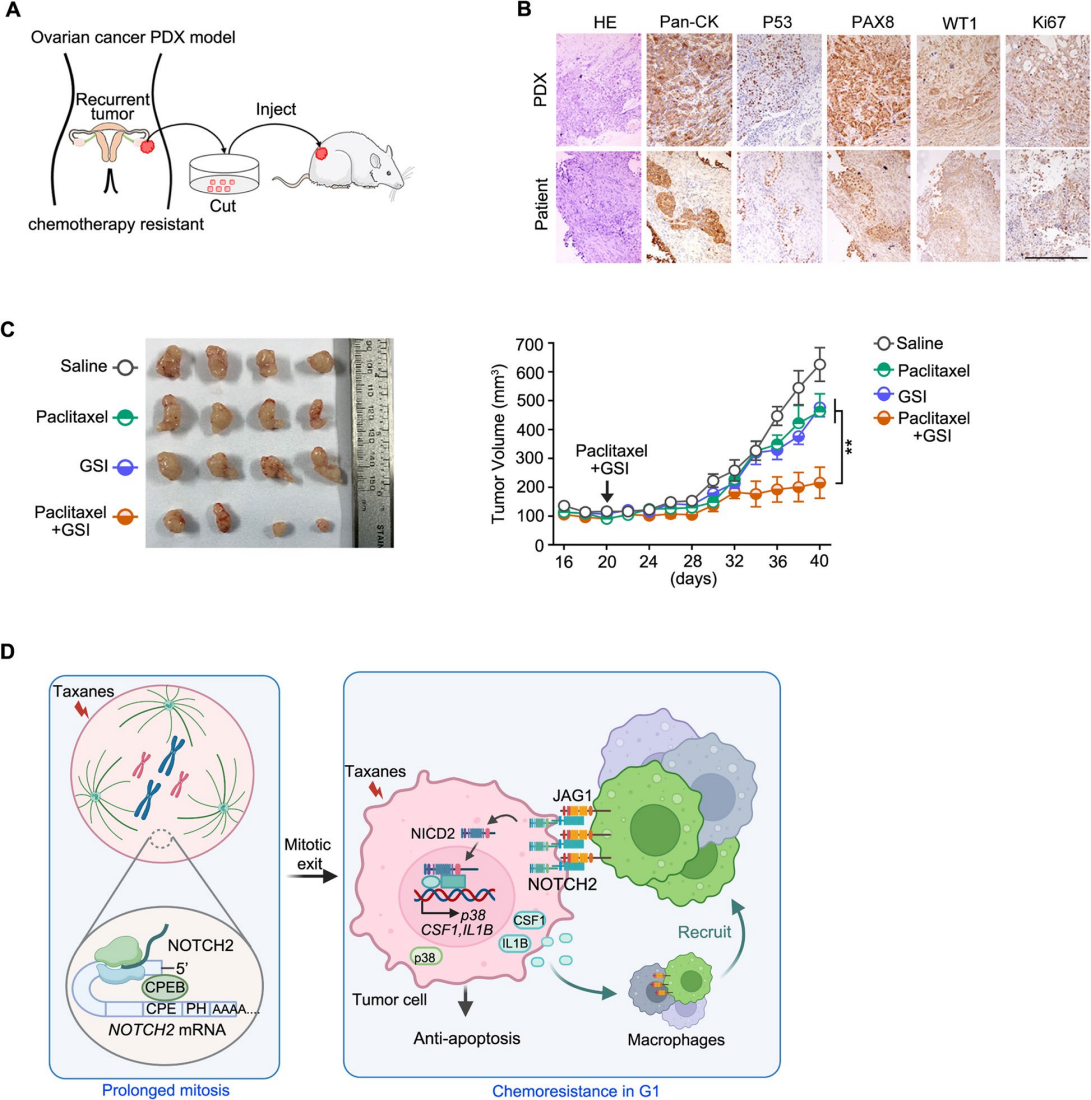
NOTCH2 inhibitor sensitizes ovarian cancer patient-derived xenograft tumor response to paclitaxel. **A** Schematic diagram of PDX model generation from ovarian cancer patient tumor samples. **B** Immunohistochemistry (IHC) staining of ovarian cancer markers in patient tumor tissues and corresponding PDX tumors. Scale bar, 100 μm. **C** Effect of paclitaxel, GSI, or their combination on tumor growth in PDX mice (*n* = 4 per group). Representative tumors (left) and tumor volumes (right) are shown (mean ± SEM). **D** Proposed working model. Our results revealed that NOTCH2 translation was upregulated during paclitaxel-induced prolonged mitosis and the NOTCH2-JAG1 juxtacrine signaling between macrophages was strongly activated in the post-mitotic G1 phase, which confers paclitaxel resistance. p values were calculated by two-tailed Student’s t-test (**p < 0.01)

To further test the efficacy of the combinatorial treatment of NOTCH inhibitor and paclitaxel in a clinically relevant model, a patient-derived xenograft (PDX) model for ovarian cancer was generated (Fig. 6A). The tumor tissues were from a patient with recurrent tumor after adjuvant chemotherapy with paclitaxel/platinum (Fig. S6A), and the PDX was validated by staining multiple ovarian cancer markers (Fig. 6B). We confirmed NOTCH2 expression in the tumor tissues of this PDX model (Fig. S6B). Upon combinatorial treatment of paclitaxel and GSI, significant synergistic effect in suppressing tumor growth was observed in the PDX tumor (Fig. 6C), and NOTCH2 protein level was further increased following paclitaxel treatment (Fig. S6B), again pointing to NOTCH2 inhibition as a promising combinatorial therapeutic strategy to reverse paclitaxel resistance in NOTCH2-positive tumors.

## Discussion

Chemoresistance remains a major challenge to durable responses in ovarian cancer and other solid tumors. Although paclitaxel is a major first-line therapy, most patients eventually relapse with drug-resistant disease [12, 34]. In this study, we identify a novel, druggable mechanism in which translational upregulation of NOTCH2 during paclitaxel-induced mitotic arrest primes tumor cells for juxtacrine activation by JAG1⁺ tumor-associated macrophages (TAMs), establishing a feed-forward loop that sustains tumor survival and promotes chemoresistance.

A key conceptual advance of our study is that prolonged mitosis can prime tumor cells for immediate pro-survival signaling after mitotic exit. Because transcription is globally repressed during mitosis, survival determinants must rely on post-transcriptional control. We show that NOTCH2 mRNA undergoes polyadenylation-enhanced translation during paclitaxel-induced arrest, resulting in rapid accumulation of NOTCH2 protein prior to G1 entry. This ensures that upon restoration of cell-cell contacts, NOTCH2 can be immediately activated by JAG1 ligands expressed on neighboring macrophages. Activated NOTCH2 rapidly translocates to the nucleus to initiate transcriptional programs that (i) reinforce tumor-intrinsic prosurvival signaling through PI3K-AKT and MAPK cascades, and (ii) remodel the tumor microenvironment by upregulating cytokines, such as CSF1 and IL-1β. This dual intrinsic-extrinsic program enables paclitaxel-arrested cells efficiently evade death, i.e., translational priming enables fast NOTCH2 activation, and cytokine release recruits and sustains JAG1⁺ macrophages that provide activating ligand to stimulate NOTCH2 signaling (Fig. 6D). Importantly, transcriptional upregulation of NOTCH2 begins only hours after mitotic exit, underscoring the temporal advantage afforded by translational control.

Beyond NOTCH2, our translatome profiling identified several other chemoresistance-associated genes that may functionally enhance NOTCH2 signaling. These chemoresistance-associated genes were co-enriched with NOTCH2 (e.g., ITGAV, ICAM1, PODXL, LAMB3, EMC1 and COPB2) and have been independently reported to modulate either adhesion-dependent juxtacrine signaling or membrane trafficking [35–39]. The data thus suggest that paclitaxel-resistant tumors may sustain NOTCH2 signaling not only by upregulating NOTCH2 itself but also by strengthening the cellular machinery that enables NOTCH2 activation.

Previous studies have shown that macrophages accumulate in the ovarian cancer microenvironment and correlate with poor prognosis largely due to their immunosuppressive and pro-tumorigenic activities [40]. In particular, M2-like macrophages promote angiogenesis, suppress T cell responses, and secrete cytokines that support tumor growth. Our findings extend this paradigm by showing that tumor-associated macrophages (TAMs) are not passive supporters, but active partners in chemotherapy resistance through direct ligand-receptor interactions. Our results reveal that JAG1 expression is enriched in TAMs after paclitaxel treatment. Functionally, JAG1⁺ macrophages engage NOTCH2 on tumor cells to trigger pro-survival signaling, and also simultaneously reinforcing their own expansion through NOTCH2-induced cytokines. This dual role establishes JAG1⁺ macrophages as both initiators and amplifiers of chemoresistance.

Single-cell RNA-seq analysis further revealed that JAG1 expression defines a distinct macrophage subset enriched for oxidative stress response, lipid metabolism, and extracellular matrix remodeling pathways, all hallmarks of metabolically adapted, immunosuppressive macrophages [41]. Notably, paclitaxel selectively enriched this JAG1⁺ subset, whereas combinatorial NOTCH inhibition depleted it. These findings underscore how chemotherapy itself reshapes the tumor immune landscape, favoring the protumor macrophage populations that in turn protect tumor cells from drug-induced death.

From a translational standpoint, our data support selective disruption of the NOTCH2-JAG1 axis as a strategy that is more tumor-specific than broad macrophage depletion. Strategies such as CSF1R blockade or clodronate liposomes can enhance chemotherapy efficacy, but they also risk impairing macrophages’ essential roles in host defense and tissue repair. By contrast, targeting NOTCH2-JAG1 signaling could specifically attenuate protumor macrophage functions, while preserving systemic macrophage activity. Importantly, NOTCH2 inhibition produces a dual therapeutic gain: attenuating intrinsic tumor cell survival while limiting macrophage infiltration and immunosuppression. This dual benefit makes NOTCH2 an attractive combinatorial target. NOTCH-targeted therapies have seen significant advancement recently [42–46]. The γ-secretase inhibitor Nirogacestat received FDA approval in 2023 for desmoid tumors, validating the clinical efficacy and feasibility of pharmacological NOTCH blockade [47]. Furthermore, receptor-specific biologics such as Tarextumab (anti-NOTCH2/3) enhanced taxane responses in breast and ovarian cancer models, although clinical translation was limited in pancreatic cancer due to toxicity and disease-specific factors [48, 49]. These observations highlight the need for receptor-specific strategies, and our results suggest that a NOTCH2-specific inhibitor or antibody, when paired with anti-mitotic drugs, may provide a superior therapeutic index in NOTCH2-positive ovarian tumors.

In this study, we analyzed JAG1 expression in malignant ascites from patients with high-grade serous ovarian carcinoma (HGSOC) because malignant ascites is a clinically accessible and abundant source of viable tumor cells and tumor-associated macrophages (TAMs), the two key cell types central to NOTCH2-JAG1 juxtacrine signaling [50]. Unlike surgical tumor biopsies that provide only a static, region-restricted view of the tumor microenvironment, ascites enables longitudinal monitoring of the therapy-driven tumor-immune remodeling for understanding chemoresistance [50, 51]. Although paired ascites and surgical tumor specimens from the same patients were not available in our study, we evaluated JAG1 expression in matched ascitic and solid tumor samples from the mouse ovarian cancer model. JAG1 expression was consistently detected in both the ascites and bulk tumor (Supplementary Fig. 3H), indicating that ascites recapitulates the key NOTCH2-JAG1 signaling characteristics of the solid tumor microenvironment. JAG1-blocking or neutralizing antibodies are currently under active therapeutic development [52]. And our results support combining such agents with paclitaxel-based chemotherapy as a rational approach to enhance chemosensitivity and delay the emergence of resistance in patients with JAG1-high ovarian tumors.

We recognize there are notable limitations of our study. While our co-culture and depletion studies support a role for JAG1⁺ macrophages, definitive evidence will require macrophage-specific JAG1 deletion or lineage-tracing models. Our in vivo pharmacological inhibition analysis relied on a pan-NOTCH, but not NOTCH2-sepcific, inhibitor. Although our genetic perturbation results suggest NOTCH2 is the key effector, contributions from other NOTCH receptors cannot be entirely excluded. Moreover, macrophage depletion in our the in vivo experiments was not subset-specific, limiting our ability to attribute resistance solely to JAG1⁺ macrophages. Finally, our clinical specimen cohort was modest, constraining the generalizability of NOTCH2 as a predictive biomarker. Future work with refined genetic models, NOTCH2-specific inhibitors, and larger patient cohorts will be necessary to validate and extend the findings.

## Conclusion

In summary, this study identifies a translationally primed, contact-dependent NOTCH2-JAG1 signaling circuit that couples paclitaxel-induced prolonged mitosis to microenvironmental adaptation and chemoresistance. By elucidating how translational regulation primes tumor cells for survival and by characterizing JAG1⁺ TAMs as specialized activators of chemoresistance, we provide the rationale basis for dual targeting of tumor-intrinsic and tumor extrinsic microenvironment pathways. Selective targeting of NOTCH2-JAG1 signaling may disrupt the tumor-macrophage interaction, sensitize tumors to taxanes, and ultimately improve treatment outcomes for patients with ovarian cancer.

## Methods

### Cell lines and cell culture

HeLa, OVCAR8, OVSAHO, ID8, HEK293T, and HeyA8 cells were purchased from the sources indicated in the Key Resources Table (Table S3). Cells were cultured in Dulbecco’s modified Eagle’s medium (DMEM; Invitrogen/Thermo Fisher Scientific, MA, USA) supplemented with 10% fetal bovine serum (FBS; Gibco, Grand Island, NY, USA) and 1% penicillin–streptomycin (P/S; Beyotime Biotechnology, Jiangsu, China) at 37 °C in a humidified atmosphere with 5% CO₂. THP-1 cells were maintained in RPMI-1640 medium (Invitrogen/Thermo Fisher Scientific) supplemented with 10% FBS and 1% P/S. All cell lines were regularly authenticated and confirmed to be free of mycoplasma contamination.

### Cell synchronization

Cancer cells were synchronized using a double-thymidine block (2 mM; Sigma). Briefly, cells were seeded in 10-cm dishes one day before treatment and incubated with 2 mM thymidine for 19 h to arrest cell cycle progression. After three washes with PBS, cells were released into fresh medium for 9 h, followed by a second thymidine block for 16 h. Cells were then washed again with PBS and released into complete DMEM. After 9 h, cells were treated with DMSO or paclitaxel (5 nM for HeLa, 10 nM for OVCAR8, and 100 nM for OVSAHO). Mitotic cells were harvested by mechanical shake-off. Cells treated with DMSO were designated as mitotic (M), and those treated with paclitaxel as prolonged mitotic (pM). Synchronization and mitotic arrest efficiency were confirmed by microscopy and flow cytometry.

### Ribosome profiling and mRNA sample Preparation

HeLa cells were synchronized by double-thymidine block (2 mM, Sigma). Mitotic (M) and prolonged mitotic (pM) cells were lysed in buffer containing 20 mM Tris (pH 7.5), 150 mM KCl, 5 mM MgCl₂, 1 mM dithiothreitol, and 8% glycerol, supplemented with 0.5% Triton X-100, 30 U/mL Turbo DNase (Ambion, Life Technologies, CA, USA), and 100 µg/mL cycloheximide (Sigma-Aldrich, MO, USA). Ribosome-protected fragments were isolated and sequenced as previously described [53]. Briefly, mRNAs were partially hydrolyzed in bicarbonate buffer to yield an average fragment size of ~ 80 bp. Fragments of 26–34 nt were separated by denaturing PAGE, and sequencing libraries were prepared and processed as described [53].

### Immunofluorescence

Immunofluorescence was conducted as previously described [54]. For cell lines, cells were plated onto 22 × 22 mm coverslips 24 h prior to treatment. Cells were washed with warmed PHEM (60mM PIPES (pH 6.8), 25mM HEPES, 10 mM EGTA and 2 mM MgCl_2_) and fixed in 4% formaldehyde for 15 min (min) at room temperature. For tumor tissues, the tissues were fixed in 4% formaldehyde for 14 h at 4 °C prior to dehydrating by 30% sucrose for 24 h at 4 °C. The tissues were then embedded into OCT gel and stored at -80 °C before dissection for slides. For human or mouse ascites, fresh ascites were centrifuged at 2000 rpm for 5 min at 4 °C to collect cells and spread on Poly-L-Lysine coated slides. 10 min later, when the ascites on slides were dried, cells were fixed in 4% formaldehyde for 15 min at room temperature. Then cells or tissues were extracted by 0.2% Triton X-100 in PHEM, blocked with 1% bovine serum albumin in TBST for 30 min, incubated with primary antibody for 2 h at room temperature, washed with TBST three times and incubated with secondary antibodies for an additional 1 h at room temperature. DNA was stained with 4, 6-diamidino-2-phenylindole (DAPI) for 3 min. Coverslips were mounted using ProLong antifade (Sigma). Images were acquired using a DeltaVision microscope (GE Healthcare, Buckinghamshire, UK) with a 60× or 20× objective lens, and optical sections were acquired 0.3–0.5 μm apart in the z-axis and shown as maximal intensity projections.

### Western blot

Cells were seeded in culture dishes 24 h before treatment. After aspiration of medium, cells were washed three times with PBS and lysed in buffer containing 50 mM Tris-HCl (pH 7.4), 250 mM NaCl, 1 mM EDTA, 50 mM NaF, 0.5% Triton X-100, and protease inhibitors. Lysates were incubated on ice for 30 min and centrifuged at 12,000 rpm for 15 min at 4 °C. Supernatants were collected, and protein concentrations were determined using a Bradford assay kit (Sangon Biotech, Shanghai, China). Equal amounts of protein were separated by SDS-PAGE, transferred onto polyvinylidene difluoride (PVDF) membranes (Millipore), and detected using specific primary and secondary antibodies. Signals were visualized with Western Lightning Chemiluminescence Reagent Plus (Advansta, Menlo Park, CA, USA).

### NOTCH2 mRNA sequence alignment

The NOTCH2 mRNA sequence was obtained from the NCBI database. We then performed sequence alignment based on the CPE and PH motifs [31] using the SnapGene software (www.snapgene.com).

### RNA Immunoprecipitation (RIP)

RIP was carried out as previously described [55]. Briefly, HeLa cells in prolonged mitosis were crosslinked in a UV crosslinker (UVP) at 120 mJ/cm² for 2 min. Cells were lysed in ice-cold RIPA buffer (50 mM Tris-HCl, pH 8.0; 150 mM NaCl; 5 mM EDTA; 1% NP-40; 1% SDS) and sonicated for 5 min using a Sonics Vibra-Cell (3-second on and 6-second off cycles). Lysates were centrifuged at 13,000 × g for 20 min at 4 °C, and supernatants were collected. Antibody or IgG control was pre-incubated with Protein G Dynabeads (Life Technologies) for 4 h at 4 °C, followed by incubation with cell lysates for ≥ 4 h at 4 °C. Protein-antibody-bead complexes were washed twice with RIPA buffer and twice with high-salt RIPA buffer (500 mM NaCl).

### Live cell imaging

For live-cell imaging, 2 × 10⁴ synchronized cells were seeded into 8-well chamber slides (#80826, ibidi) and cultured overnight at 37 °C in DMEM supplemented with 10% FBS and 1% penicillin–streptomycin (P/S). The medium was then replaced with phenol red-free L-15 medium (Invitrogen/Thermo Fisher Scientific, MA, USA) containing 10% FBS and 1% P/S. Time-lapse imaging was performed at 5-min intervals using a 20× objective lens on an Eclipse Ti microscope (Nikon, Tokyo, Japan). Mitosis was defined morphologically by cell rounding, and interphase by flattened morphology. Cell death was identified based on characteristic morphological changes, including membrane blebbing and cell lysis.

### Gene knockdown by ShRNA

Lentiviral particles were generated in HEK293T cells and harvested 48 h after transfection. Viral supernatants were filtered through 0.45-µm non-pyrogenic filters (Merck Millipore, Billerica, MA, USA). For infection, target cells were seeded at 1–3 × 10⁵ cells per 35-mm dish. After 24 h, cells were incubated with diluted lentivirus in the presence of 8 µg/mL polybrene (Sigma-Aldrich, St. Louis, MO, USA). Viral medium was replaced with fresh culture medium 24 h post-infection (48 h for THP-1 cells). Stable transductants were selected with 1 µg/mL puromycin beginning 48 h after infection.

### CRISPR-Cas9 gene knockout

One pair of guides against the exon-30 sequence of Human NOTCH2 was designed using the guide design tool found at: (https://zlab.bio/guide-design-resources/) with the input of Human NOTCH2 exon-30 sequence. Guide sequences were cloned into the pSpCas9 (BB)-2 A-Puro vector that was previously described [56]. Lipo3000 was used to transfect the cells, and puromycin was used to select the transfected cells. Cells were selected for five days after transfection, then plated for single colonies. Multiple candidate clones were picked and tested for gene disruption by western blot. The ones in which NOTCH2 protein expression was downregulated were selected for genomic extraction and PCR. The PCR fragment was sent to SANGON for sequencing to confirm that NOTCH2 exon-30 was disrupted. The CRISPR-guide sequences and vector information were listed in the Key Resources Table.

### Soluble Recombinant rJAG1 ligand immobilization and cell stimulation

Recombinant rat rJAG1-Fc fusion protein, goat anti-human IgG Fc antibody, and recombinant human IgG1-Fc (R&D Systems, Minneapolis, MN, USA) were dissolved in PBS at a final concentration of 10 µg/mL. Immobilization of rJAG1-Fc and the IgG-Fc control was performed as previously described [57, 58]. Briefly, 2 µg (for 12-well plates) or 0.5 µg (for 8-well chamber slides) of goat anti-human IgG Fc antibody was added per well and incubated for 2 h at room temperature. Wells were aspirated and washed three times with PBS, followed by addition of 1 µg (12-well plate) or 0.25 µg (8-well chamber slide) recombinant rJAG1-Fc or human IgG1-Fc. Plates or slides were incubated at 4 °C for 20 h, washed with PBS, and subsequently seeded with 1 × 10⁵ or 1 × 10⁴ cells per well, respectively.

### RNA-Seq and KEGG pathway enrichment analysis

Total RNA was extracted from OVCAR8 cells using TRIzol reagent (Invitrogen, Carlsbad, CA, USA) following the manufacturer’s instructions. RNA concentration and quality were assessed using a NanoDrop spectrophotometer and an Agilent 2100 Bioanalyzer (Thermo Fisher Scientific, MA, USA). RNA integrity, library preparation, and sequencing were performed by the Beijing Genomics Institute (BGI, Shenzhen, China). Briefly, mRNA was purified using oligo(dT)-conjugated magnetic beads and fragmented with fragmentation buffer at an appropriate temperature. cDNA synthesis was carried out according to the manufacturer’s protocol, and quality was validated using the Agilent 2100 Bioanalyzer. DNA nanoballs (DNBs) were generated by phi29 amplification, loaded onto patterned nanoarrays, and sequenced on the BGISEQ-500 platform to produce single-end 50 bp reads.

Sequence reads were aligned to the human reference genome GRCh38.p11 (hg38). Differentially expressed genes (DEGs) were identified using the DESeq2 R package, with adjusted *p* < 0.05 and log₂ fold change < − 0.5 as cutoffs. A total of 522 significantly downregulated genes were obtained from overlapping results of two comparisons (NOTCH2-sh1 vs. NTC and NOTCH2-sh2 vs. NTC). KEGG pathway enrichment analysis was conducted using KOBAS 3.0 (http://kobas.cbi.pku.edu.cn/kobas3/genelist/), with false discovery rate (FDR) < 0.05 as the threshold for significant enrichment. RNA-seq data have been deposited in the NCBI Gene Expression Omnibus (GEO) under accession number GSE158569.

### Gene expression analysis by qRT-PCR

qRT-PCR was conducted as previously described [59]. Briefly, total RNA was extracted using TRIzol reagent (Invitrogen, Carlsbad, CA, USA) according to the manufacturer’s protocol, and RNA concentration was determined with a NanoDrop spectrophotometer (Life Technologies, Carlsbad, CA, USA). cDNA was synthesized from 1 µg RNA using oligo(dT) primers and Superscript III reverse transcriptase (Vazyme, Nanjing, China). Quantitative PCR was carried out using SYBR^®^ Premix (Vazyme, Nanjing, China) on a PikoReal 96 real-time PCR system (Thermo Scientific, MA, USA). All reactions were run in triplicate. Primer sequences used in this study are listed in the Key Resources Table (Table S3). Primers were designed using PrimerBank or OriGene (OriGene Technologies, Rockville, MD, USA) and specificity was confirmed with NCBI Primer-BLAST. Oligonucleotides were synthesized by Sangon Biotech (Shanghai, China).

### ELISA

ells were seeded in 60-mm dishes with 3 mL complete medium 24 h before treatment. After the indicated treatments for 72 h, conditioned media were collected, centrifuged at 1000 × g for 10 min, and supernatants were harvested. IL-1β and CSF1 levels in the supernatants were quantified using ELISA kits (ABclonal, Wuhan, China) according to the manufacturer’s instructions. Cytokine concentrations were normalized to cell numbers.

### Generation of THP-1- or PBMCs-derived macrophages and the co-culture assay with cancer cells

M2-like THP-1 macrophages were generated as described previously [60]. Briefly, 2 × 10⁶ THP-1 cells were seeded in 35-mm dishes and treated with 320 nM PMA for 6 h, followed by culture with PMA plus 20 ng/mL IL-4 and 20 ng/mL IL-13 for an additional 18 h. Peripheral blood mononuclear cells (PBMCs) were isolated from human blood by density gradient centrifugation. After washing, PBMCs were seeded into T175 flasks in PBS and incubated at 37 °C for 1 h to allow monocyte adherence. Non-adherent cells were removed, and adherent monocytes were cultured in RPMI medium supplemented with 20 ng/mL GM-CSF to generate M0 macrophages. Medium was refreshed on day 3, and differentiation continued for a total of 6 days. M0 macrophages were detached by PBS-EDTA incubation at 37 °C for 20 min, followed by gentle tapping and scraping. Collected cells were counted, seeded into appropriate plates, and allowed to adhere. For polarization, M0 macrophages were treated with 100 ng/mL LPS plus 20 ng/mL IFN-γ for 24 h to induce M1-like macrophages, or with 20 ng/mL IL-4 for 48 h to induce M2-like macrophages.For co-culture assays, OVCAR8-GFP stable cells were seeded with M2-like macrophages at a 1:1 ratio in 6-well plates, and paclitaxel was added 24 h later. OVCAR8 cell death was assessed by Annexin V staining followed by flow cytometry.

### In vivo mouse tumor models

All animals were maintained under specific pathogen-free conditions with unrestricted access to food and water. All mouse experiments were approved by the Ethics Committee of the University of Science and Technology of China (2019-N(A)-059). To establish xenograft model, BALB/c nude mice (athymic nu/nu, 6–8 weeks old; SLAC Laboratory Animal, Shanghai, China) were subcutaneously injected with 5 × 10⁵ OVCAR8 cells. Paclitaxel was administered intraperitoneally (i.p.) at 10 mg/kg when tumors reached ~ 100 mm³ (approximately one week after injection) and continued for three weeks. For NOTCH2 knockdown experiments, OVCAR8 cells stably expressing doxycycline-inducible NOTCH2-shRNA were injected, and mice were provided doxycycline (1 mg/mL) in drinking water throughout the experiment. For inhibitor studies, mice were randomized into groups when tumors reached 100–150 mm³. Paclitaxel (10 mg/kg, i.p.) was administered every two days, and RO4929097 (3 mg/kg) was given orally every two days. Tumor size was measured using calipers, and volume was calculated as 4π/3 × (width/2)² × (length/2). Tumors were harvested after two weeks of treatment (experimental endpoint).

To establish syngeneic ID8 model, female C57BL/6 mice (8–10 weeks old; SLAC Laboratory Animal, Shanghai, China) were injected i.p. with 5 × 10⁶ ID8-p53 wild-type cells or 5 × 10⁵ ID8-p53⁻/⁻ cells. For the ID8-p53 wild-type model, ascitic fluid was collected six weeks after inoculation for immunostaining.

To generate patient-derived xenograft (PDX) model, fresh tumor tissues were obtained from patients with histologically confirmed serous ovarian cancer at the First Affiliated Hospital of USTC, with informed consent and institutional review board approval. Non-necrotic malignant tissue was dissected, minced on ice, and mixed 1:1 with Matrigel (Corning). Approximately 0.3–0.5 cm³ of tumor slurry was implanted subcutaneously into female NOD-SCID mice (≥ 3 mice per sample). Moribund mice were sacrificed, and tumors were expanded by a single passage into additional mice to generate sufficient material for banking and experiments. Tumor grafts were cryopreserved by mincing and freezing in 90% FBS and 10% DMSO at − 80 °C overnight, followed by long-term storage in liquid nitrogen.

### Macrophage depletion in the in vivo mouse tumor models

Macrophages in the C57BL/6 mice were depleted using clodronate-containing liposomes according to a published protocol [61]. Mice were intravenously injected one dose of clodronate-liposomes (0.2 mL/mouse at 5 mg/mL, From Vrije Universiteit Amsterdam) every three days and three days before Paclitaxel and GSI treatment. This treatment route and schedule were selected to target the peritoneal macrophages more effectively, including those residing in the omentum.

### Flow cytometry analysis (FACS) of Ascites

Ascites were collected and incubated in Red Blood Cell Lysis Buffer (Beyotime Biotechnology) for 10–15 min at 4 °C to lyse the red blood cells. Cells were washed and counted using automated cell counter (Countstar). 1 × 10^6^ cells were blocked with FcR Blocking Reagent (BioLegend) or mouse serum, and stained for cell surface markers. All antibodies were purchased from BioLegend. Flow cytometry acquisition was performed on CytoFLEX (BD Biosciences), and data were analyzed using CytExpert software (2.4.0.28). Changes in the proportion of tumor-infiltrating immune cell subsets were compared by dimensionality reduction using t-SNE analysis using R (version 4.3.0).

### Single-cell RNA sequencing (scRNA-seq) analysis

Single-cell RNA sequencing (scRNA-seq) was performed using the MobiCube High-throughput Single Cell 3’ Transcriptome Set V2.1 (PN-S050200301) in combination with the MobiNova-100 microfluidic platform. Tissue samples were first dissociated into single-cell suspensions using the MobiSoultion™ Tissue Dissociation Kit, and the cell concentration was adjusted to 700–1200 cells/µL. The suspension was immediately loaded onto a microfluidic chip and processed using the MobiNova-100 system to generate microdroplets. This microfluidics-based platform enables high-throughput single-cell encapsulation by combining individual cells with barcoded beads in water-in-oil droplets. The barcoded beads, cells, reagents, and oil streams are introduced into separate microchannels. Upon emulsification, each droplet ideally contains a single cell and a single bead, based on the Poisson distribution, enabling accurate single-cell capture and mRNA tagging. Following droplet formation, reverse transcription was carried out to generate cDNA, which was then amplified and used for sequencing library construction according to the manufacturer’s instructions. The resulting next-generation sequencing (NGS) libraries were sequenced by OE Biotech Co., Ltd. (Shanghai, China).

Raw FASTQ files were then processed using MobiVision software (v3.2) to perform alignment to the human reference genome, UMI counting, and generation of expression matrices. Downstream analysis was conducted using the Seurat R package, including quality control, normalization, dimensionality reduction (PCA and UMAP), clustering (Louvain algorithm), and identification of highly variable genes. Cells were filtered based on UMI counts, gene counts, and mitochondrial/ribosomal gene content, with thresholds adjusted per sample. Doublets were detected and removed using the Scrublet algorithm. Cell cycle scores were calculated to evaluate and adjust for potential confounding effects. Differential gene expression, Gene Ontology (GO), and KEGG pathway enrichment analyses were performed to characterize cluster-specific functions. Cell types were annotated using SingleR and SCINA, based on canonical marker genes and reference transcriptomic datasets. Visualization was achieved through UMAP plots, heatmaps, violin plots, and bubble charts.

Tissue distribution of clusters, we calculated the Ro/e for each cluster in different tissues to quantify the tissue preference of each cluster as previous described [62].

### Histology and immunohistochemistry (IHC)

Tissue specimens from mice were fixed in 10% buffered formalin for 24 h and stored in 70% ethanol until paraffin embedding. 5 μm sections were stained with hematoxylin and eosin (HE) or used for immunohistochemical studies. Human OV tissue samples were obtained from the First Affiliated Hospital of USTC. All samples were obtained with informed consent, and the study was approved by the Ethics Committee of USTC (2019-N(H)-212), as well as the principles expressed in the Declaration of Helsinki. Patients’ characteristics are listed in Table S2.

Immunohistochemistry was performed on formalin-fixed, paraffin-embedded mouse and human tissue sections using a biotin-avidin method as described before [59]. Sections were developed with DAB and counterstained with hematoxylin. Images were taken using a Nikon Ti microscope equipped with a 20× lens. Quantification of NOTCH2 IHC chromogen intensity was performed as previously described [63]. Briefly, staining intensity (0, 1, 2, 3) and percentage of NOTCH2 positive cells among cancer (0–25% recorded as 1, 25–50% as 2, 50–75% as 3 and > 75% as 4) were evaluated by a pathologist in USTC. The final immunoreactive scores of NOTCH2 expression were calculated by multiplying the two numbers as described before [63]. Multiplexed IHC (mIHC) analysis to examine CD68 and JAG1 expression in human OV tissue samples was conducted according to the protocol of DendronFluor TSA kit (Jilin Histova Biotechnology company, China).

### Survival curve

Kaplan–Meier survival curves were generated to assess the association between NOTCH2 expression and the progression-free survival time. Patients were stratified into high and low NOTCH2 expression groups using an optimal cut-off determined by the surv_cutpoint function in the R package *survminer*. Differences between the progression-free survival curves were evaluated using the log-rank test.

### TCGA and CNHPP data analysis

Clinical data and RNA expression data with normalized RSEM values of the serous ovarian cancer dataset (TCGA-OV) was retrieved from FireBrowse (http://firebrowse.org). 343 ovarian cancer patients had associated comprehensive follow-up records, RNA expression data and records of clinical treatment. NOTCH receptors and its ligand expression levels were compared using RSEM values. Proteomics data for ovarian cancer were obtained from TCGA. Expression levels of NOTCH receptors and ligands were extracted and analyzed using R package and GraphPad software.

### Statistical analysis

All the data analyses were performed with GraphPad Prism statistical software. p value was determined by two-way ANOVA for tumor growth, or Log-rank test for survival, or two-tailed t-tests for other analyses. A value of *p* < 0.05 was considered statistically significant.

### Ethics approval statement and patient consent statement

All mouse experiments were approved by The Ethics Committee of the University of Science and Technology of China (2019-N (A)-059). The study using patient samples was approved by the Ethics Committee of USTC (2019-N (H)-212). We have obtained the patients consent for the studies.

### Resource availability

#### Lead contact

Further information and requests for resources and reagents should be directed to and will be fulfilled by the lead contact, Zhenye Yang (zhenye@ustc.edu.cn).

#### Data and code availability

Ribosome profiling and RNA sequencing data generated in this study are available at the NCBI-GEO database with the project accession ID GEO: GSE158569 and PRJNA1338849. The Cancer Genome Atlas (TCGA) data were downloaded from Firebrowse (http://firebrowse.org/).

## Supplementary Information

Supplementary Material 1.

Supplementary Material 2.

Supplementary Material 3.

Supplementary Material 4.

Supplementary Material 5.

## Data Availability

Ribosome profiling and RNA sequencing data generated in this study are available at the NCBI-GEO database with the project accession ID GEO: GSE158569 and PRJNA1338849. The Cancer Genome Atlas (TCGA) data were downloaded from Firebrowse ( [http://firebrowse.org/](http:/firebrowse.org)).
