# Supplementary material for: Tumor-associated macrophages promote chemoresistance to Paclitaxel via activating NOTCH2-JAG1 juxtacrine signaling
Source: Mol Cancer. 2026 Jan 10;25:135. doi: 10.1186/s12943-025-02546-w (PMC13191955; doi:10.1186/s12943-025-02546-w)

Figure 1

E

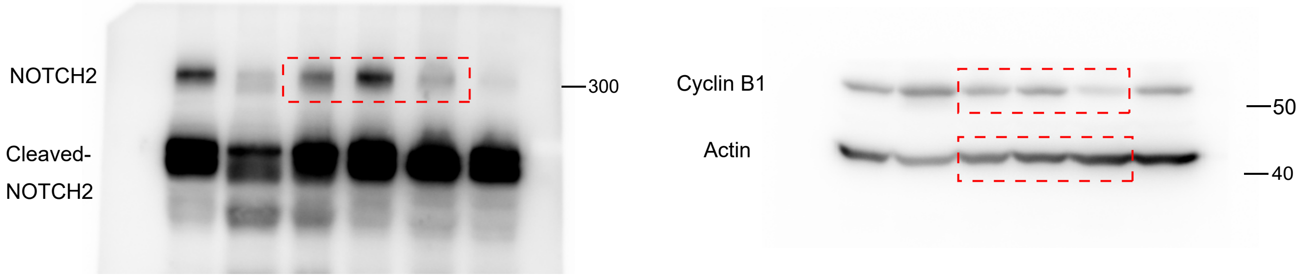

F

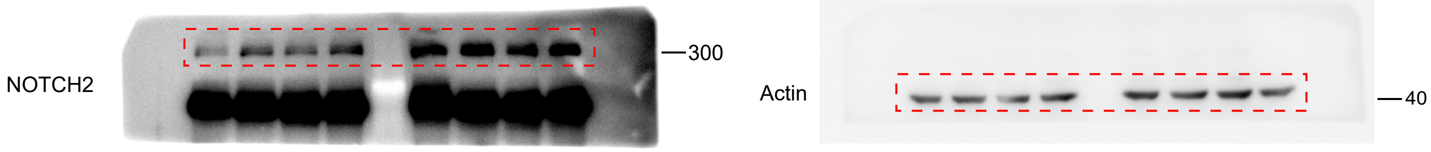

Figure S1

G

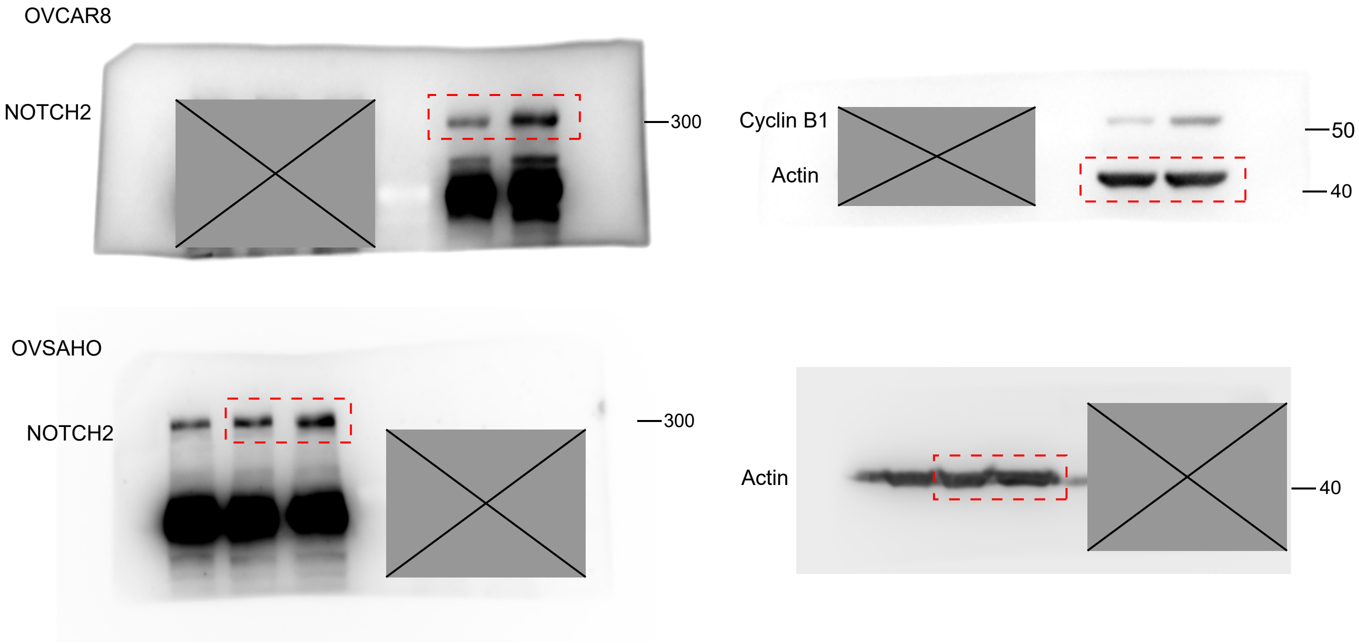

H

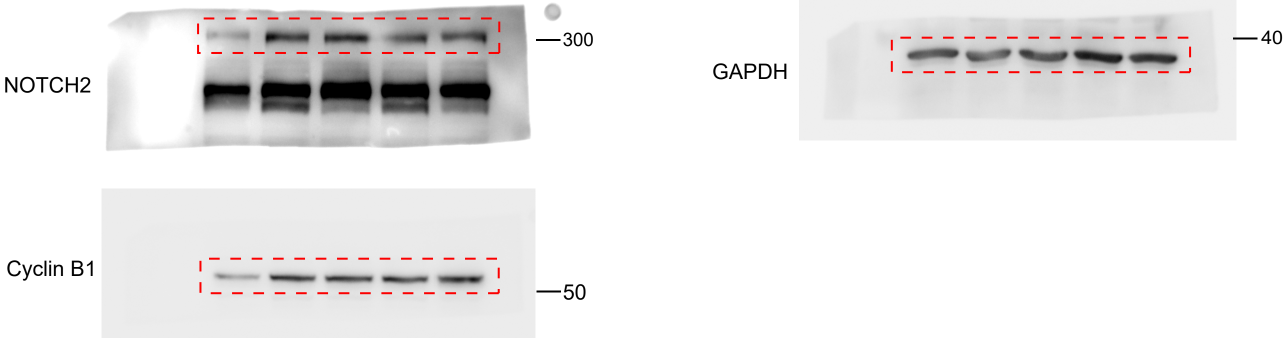

Figure 2

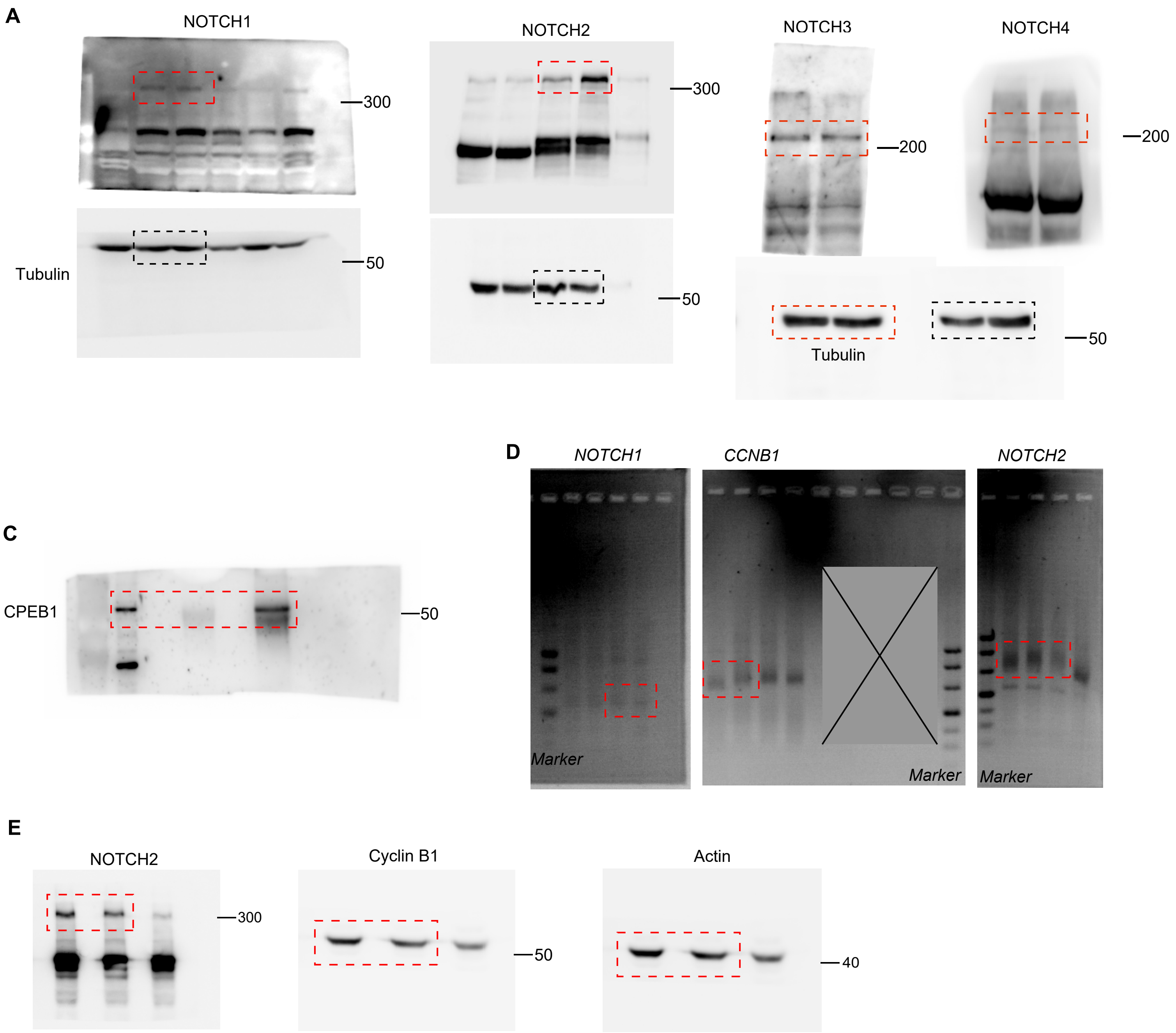

Figure S2

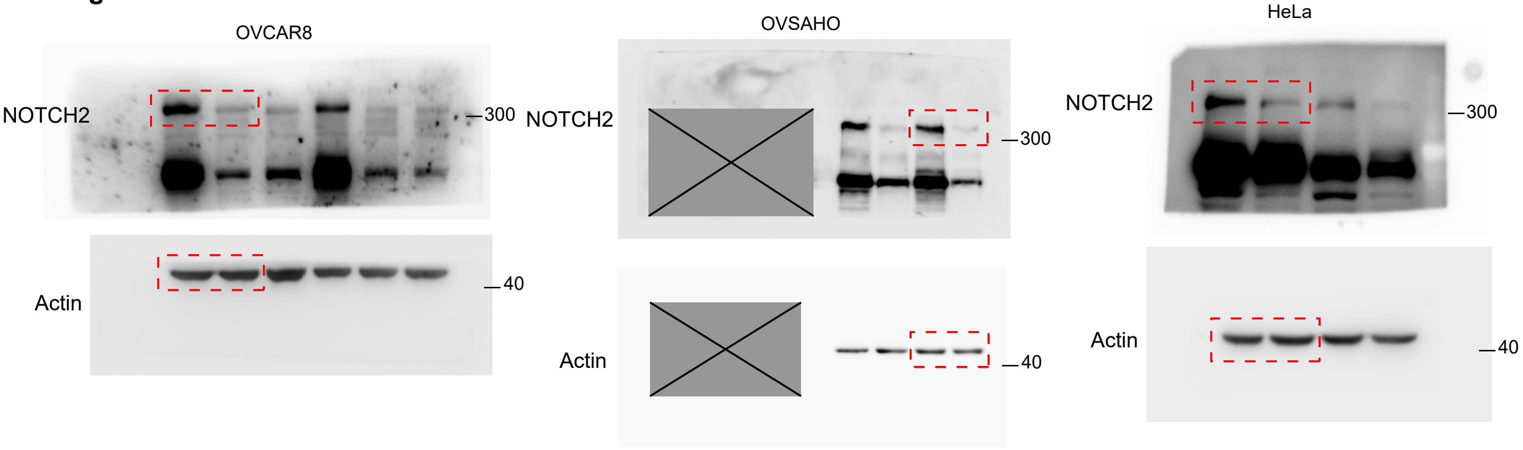

Figure 3

D

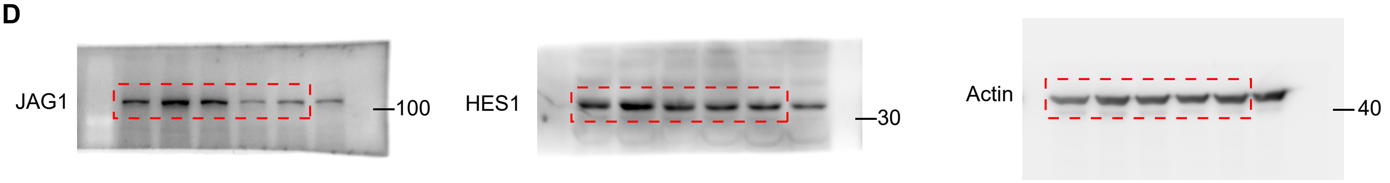

Figure S3

B

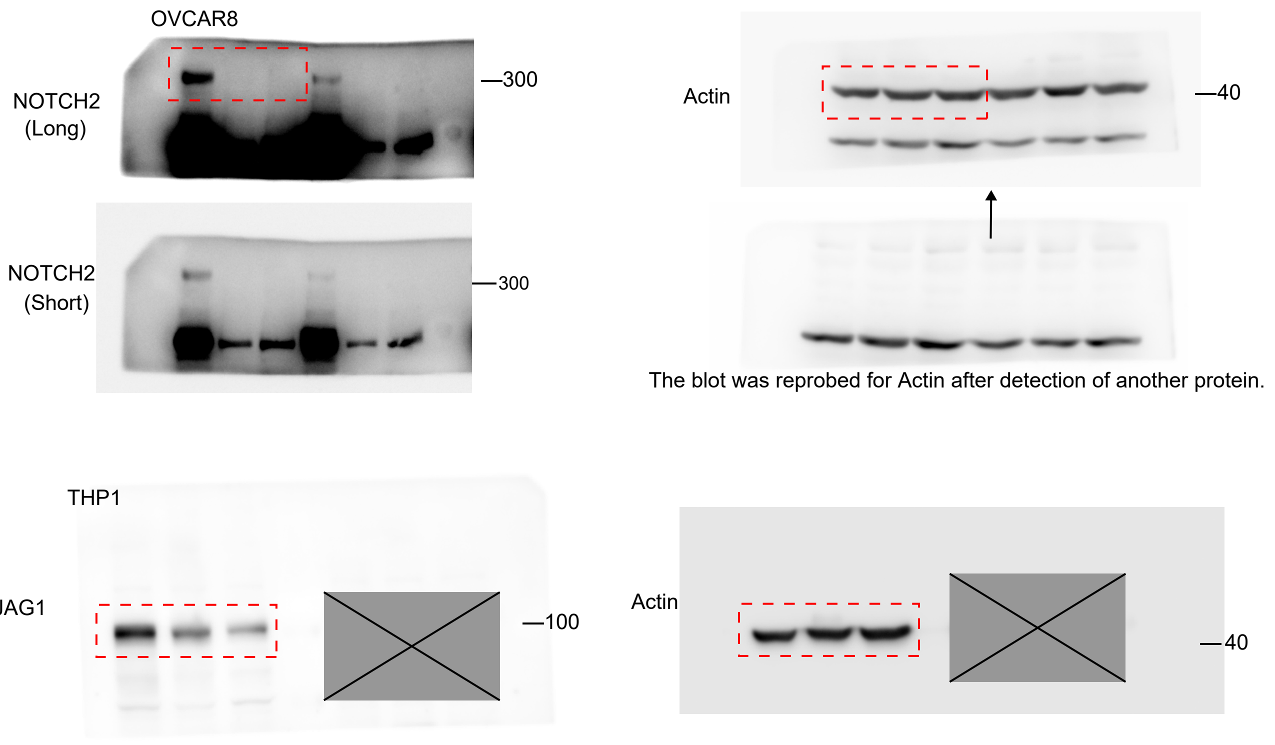

E

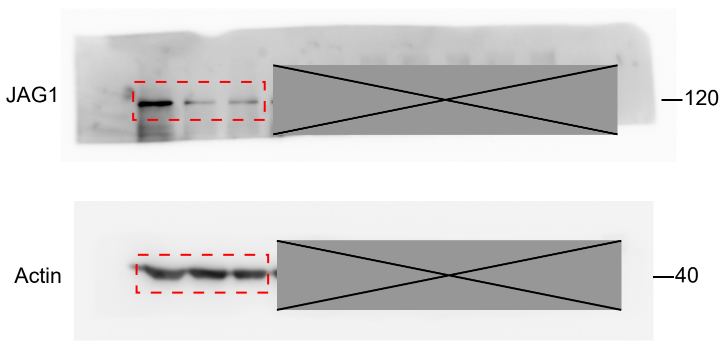

Figure S4

D

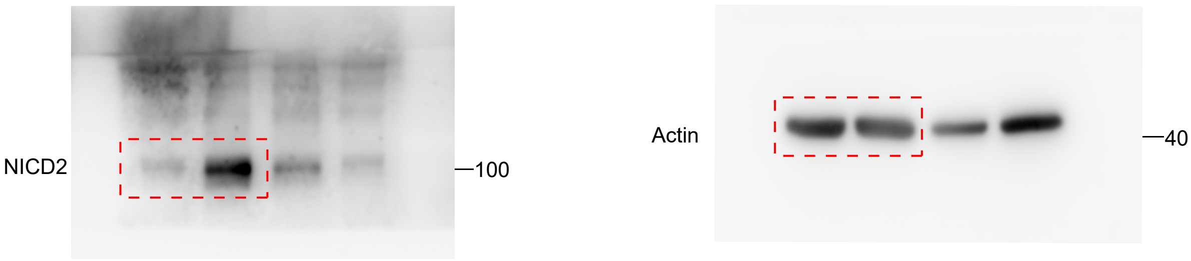

G

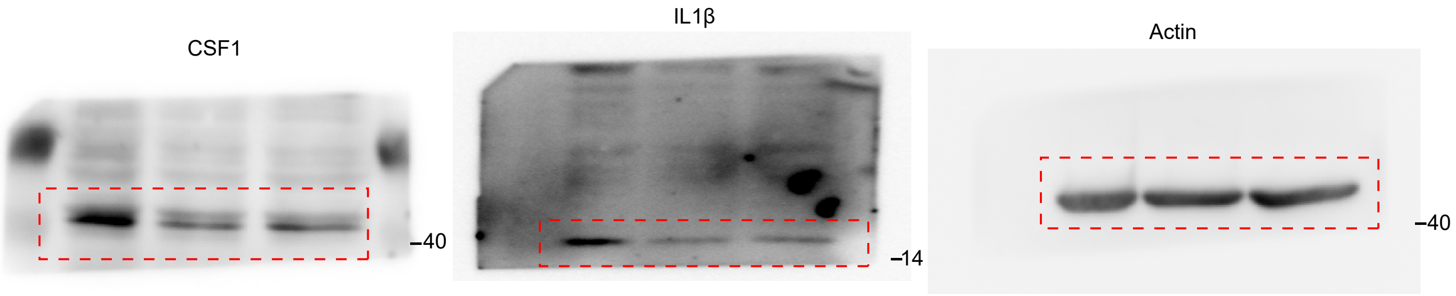

H

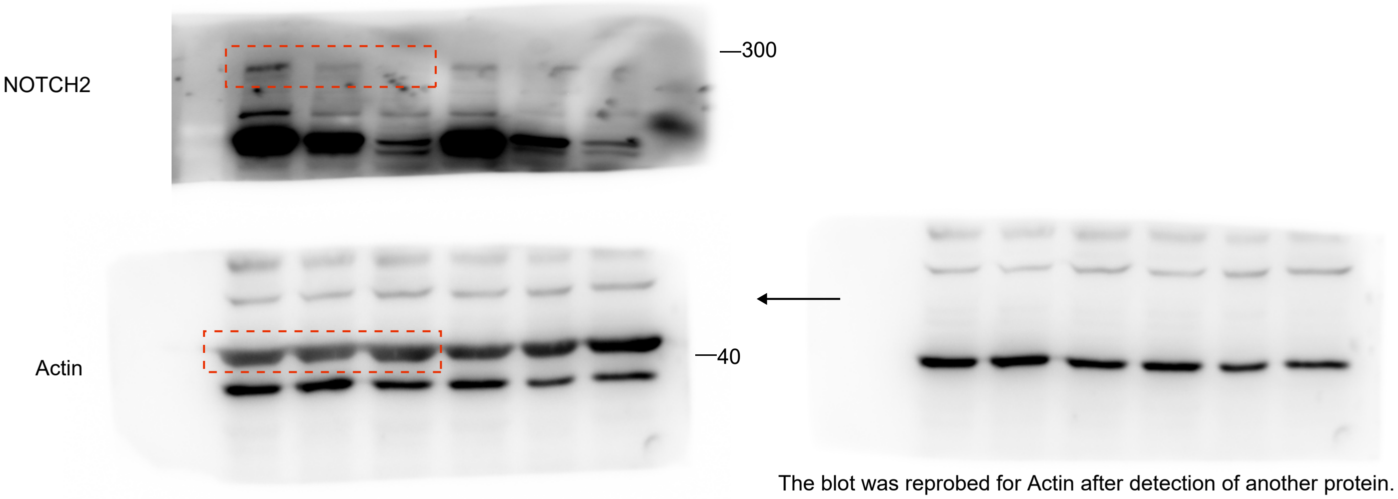

The blot was reprobed for Actin after detection of another protein.

Figure S5

A

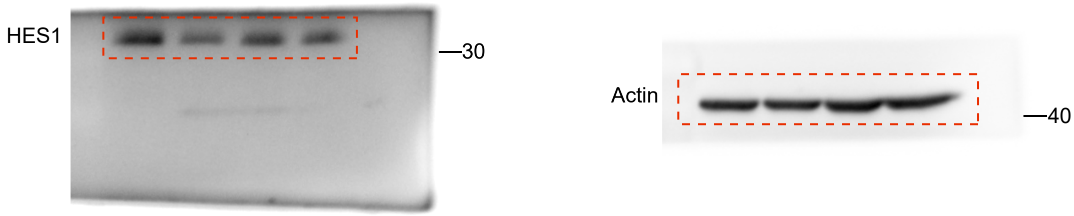

Supplement: Supplementary file 3 — Supplementary Material 3. [file 12943_2025_2546_MOESM3_ESM.pdf]
