# Supplementary material for: Tumor-associated macrophages promote chemoresistance to Paclitaxel via activating NOTCH2-JAG1 juxtacrine signaling
Source: Mol Cancer. 2026 Jan 10;25:135. doi: 10.1186/s12943-025-02546-w (PMC13191955; doi:10.1186/s12943-025-02546-w)
Supplement: Supplementary file 5 — Supplementary Material 5. [file 12943_2025_2546_MOESM5_ESM.docx]

**Supplemental information**

**Targeting NOTCH2-JAG1 juxtacrine signaling reverses macrophage-mediated tumor resistance to paclitaxel**

Fazhi Yu, Qin Zhou, Weiqiang Yu, Tong Zhou, Cheng Cao, Yijia Xie, Peng Zhang, Wei He, Aoxing Cheng, Hanyuan Liu, Qingfa Wu, Xiaopeng Ma, Jing Guo, Ying Zhou, Jue Shi, Zhenye Yang


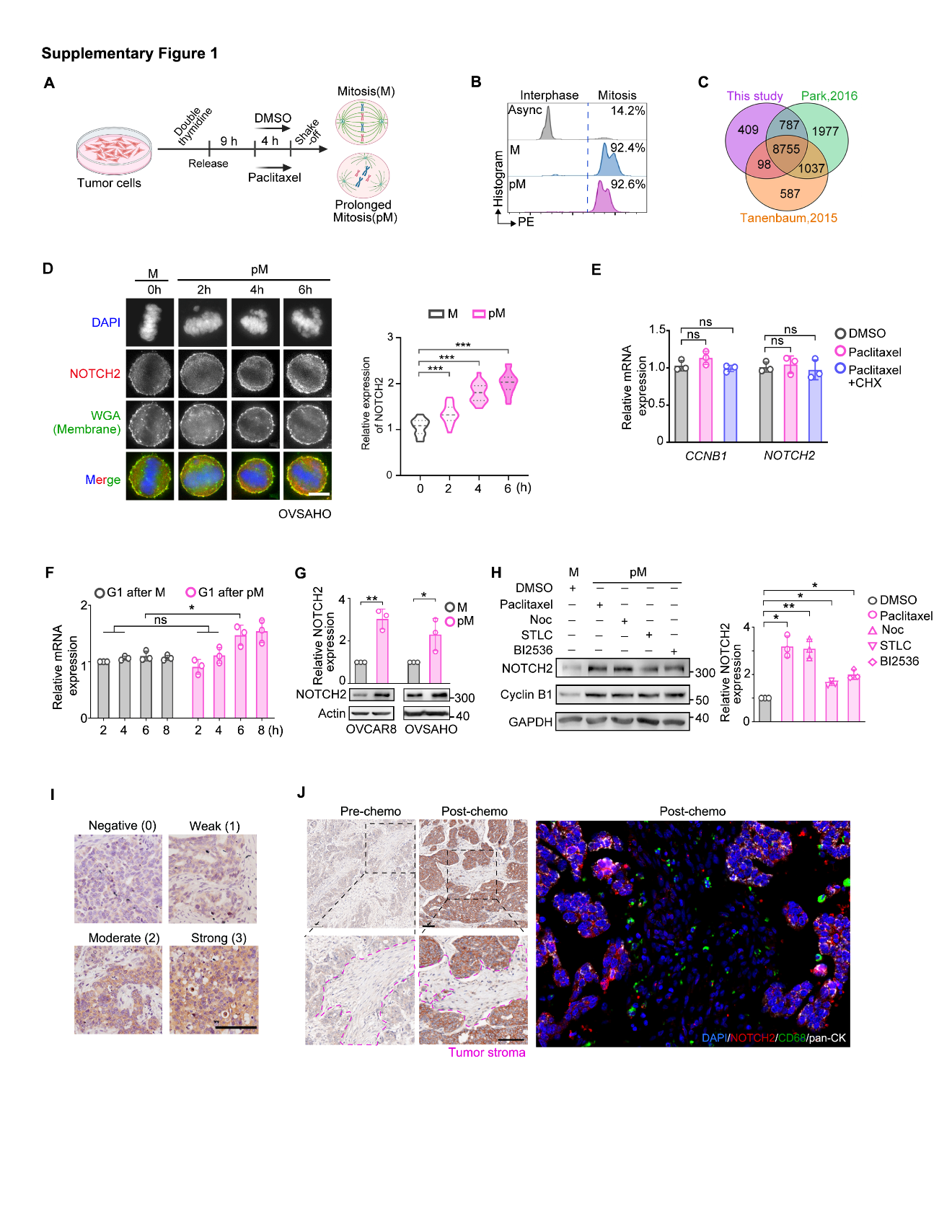
**Figure S1**. **NOTCH2 translation is upregulated during paclitaxel-induced prolonged mitosis, related to Figure 1. (A)** Workflow of mitotic and prolonged mitotic cells preparation. **(B)** Flow cytometry was used to detect cell cycle distribution of asynchronized and synchronized HeLa cells. **(C)** Venn diagram showing the overlapping genes protected by ribosome during mitosis observed in this study, Park et al. (2016)^S1^, and Tanenbaum et al. (2015)^S2^. (**D**) OVSAHO cells were synchronized, and the mitotic cells were re-plated to coverslips after shake-off. Immunofluorescence was used to detect the expression levels of NOTCH2 in OVSAHO cells in mitosis (M) and prolonged mitosis (pM) induced by paclitaxel, followed by quantitative analysis. Scale bar, 10 mm. **(E)** RT–PCR detection of mRNA levels of the indicated genes in HeLa cells in mitosis (M) and prolonged mitosis (pM). **(F)** HeLa cells in mitosis (M) and prolonged mitosis (pM) induced by 0.5 μM Nocodazole were harvested by shake-off and re-plated in a culture dish with fresh medium for various hours before being collected. RT-PCR were conducted to analyze the mRNA levels of *NOTCH2*. **(G)** Western blot analysis of NOTCH2 in OVCAR8 and OVSAHO cells in mitosis (M) and prolonged mitosis (pM). Quantified results are shown at the top. **(H)** Western blot analysis of NOTCH2 in HeLa cells in mitosis (M) and prolonged mitosis (pM). Cells in prolonged mitosis were induced by 5 nM paclitaxel, 0.5 μM Nocodazole (Noc), 5 μM STLC and 100 nM BI2536, respectively. **(I)** Representative IHC staining images of tumor specimens from ovarian cancer patients. Staining intensity of 0 (negative), 1 (weak), 2 (moderate), and 3 (strong) of NOTCH2 are shown, Scale bar=100mm. (J) Representative immunohistochemical staining of NOTCH2 in paired tumor specimens from ovarian cancer patients collected before and after chemotherapy. Stromal regions are delineated by purple dotted lines. Scale bars, 200 μm. All patients received standard adjuvant carboplatin-paclitaxel treatment. Multicolor immunofluorescence staining for NOTCH2, CD68, and pan-cytokeratin (pan-CK) was additionally performed on post-chemotherapy tumor tissues. Data are shown as mean ± SD, and data of (B), (D), (E), (F), (G) and (H) were from three experimental replicates. p values were determined by two-tailed Student’s t test (ns, not significant; *p < 0.05, **p < 0.01).


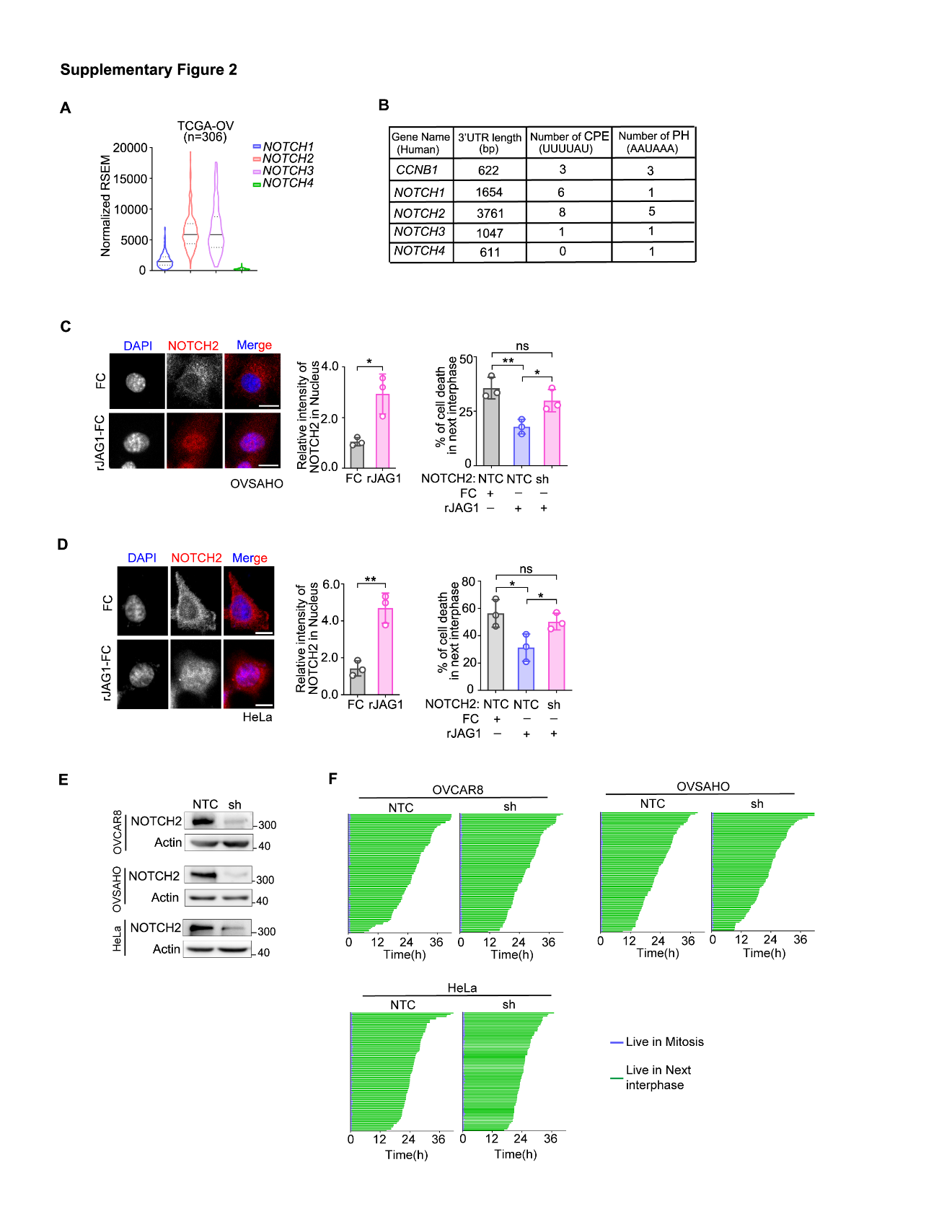


**Figure S2.** **Polyadenylation-enhanced NOTCH2 translation during prolonged mitosis confers resistance to cell death in the subsequent interphase, related to Figure 2. (A)** Analysis of NOTCH receptors at the mRNA level in ovarian cancer using TCGA data. **(B)** Characteristics of the 3’-UTR of the *NOTCH* receptor and *CCNB1* transcripts. The length and number of cytoplasmic polyadenylation elements (CPE) and polyadenylation hexanucleotide (PH) are presented. **(C-D)** Immunofluorescence showing nuclear accumulation of NOTCH2 upon rJAG1 stimulation in (C) OVSAHO and (D) HeLa cells. Cells were plated on Fc or rJAG-1 Fc for 12 h, and NOTCH2 was analyzed using immunofluorescence. Scale bar, 10 μm. Quantification of NOTCH2 fluorescence intensity in cell nucleus is shown using relative fluorescence units. **(E)** NOTCH2 expression detected in the wild-type and NOTCH2-deficient cells. **(F)**Cell cycle profile comparison based on live-cell imaging results. Data are shown as mean ± SD, and data of (C), (D) and (F) were obtained from at least three experimental replicates. p values were determined by two-tailed Student’s t test (ns, not significant; *p < 0.05, **p < 0.01).


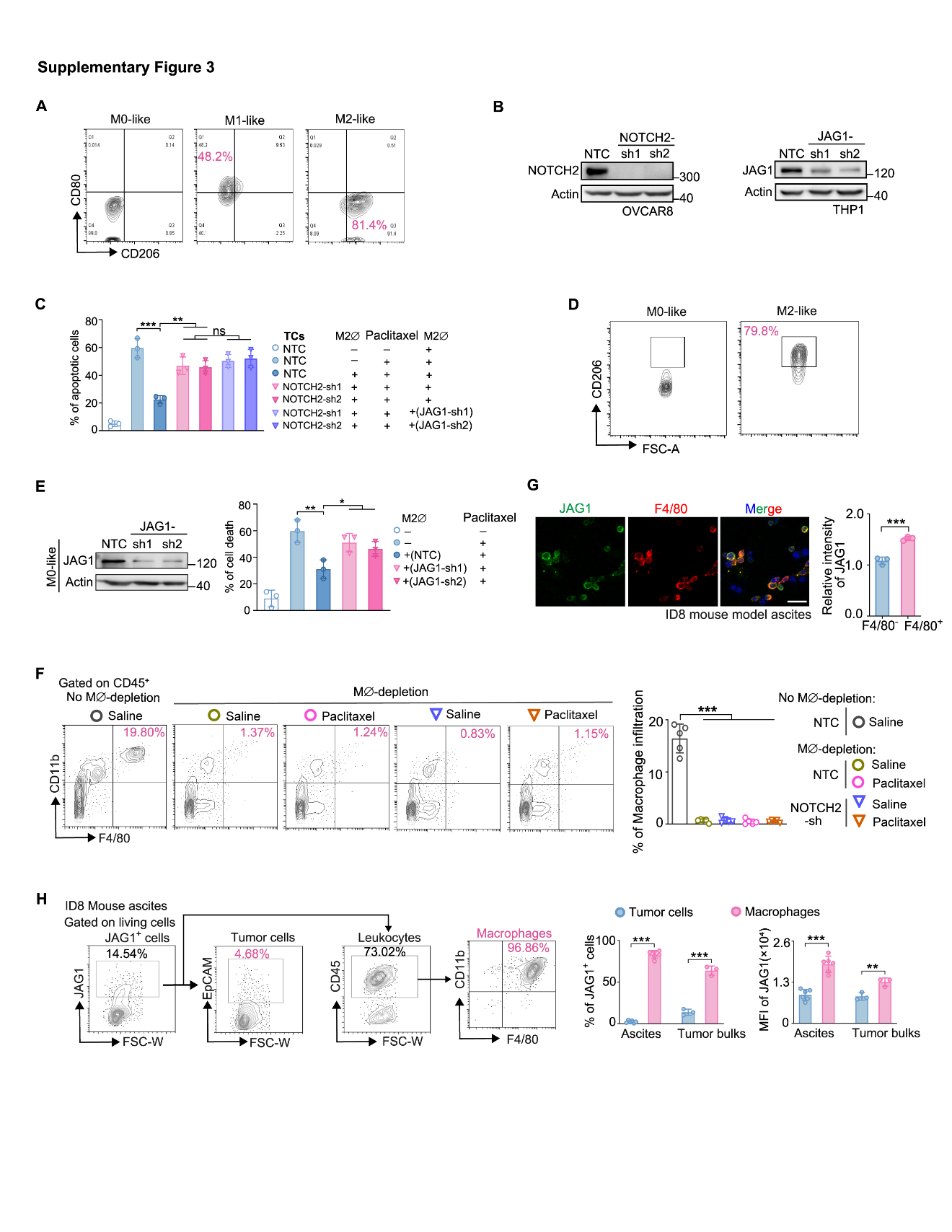


**Figure S3**. **JAG1 expressed on the macrophages is primarily responsible for activating NOTCH2 signaling and promoting paclitaxel resistance *in vivo*, related to Figure 3. (A)** The percentage of M1-like and M2-like macrophages derived from THP-1, quantified by flow cytometry. **(B)** Western blot analysis of the knockdown efficiency of NOTCH2 and JAG1 in OVCAR8 and THP-1 cells, respectively. **(C)** OVCAR8-GFP cells was co-culture with THP-1-derived M2-like macrophages and OVCAR8-GFP cell death was quantified by flow cytometry analysis of Annexin V staining. **(D, E)** Co-culture assay of GFP-positive OVCAR8 cells with PBMC-derived M2-like-macrophage. The percentage of M2-like macrophages was quantified by flow cytometry(D). And OVCAR8-GFP cell death was quantified by flow cytometry analysis of Annexin V staining (E). **(F)** Percentage of infiltrated macrophage in the ID8 mouse ascites in Figure 3F. **(G)** Immunofluorescence images (left panel) and the quantified intensity of JAG1 (right panel) expressed on macrophages in the ID8 tumor ascites. Three ascites were analyzed for each group. Scale bars, 10 mm. **(H)** Flow cytometry analysis of the expression of JAG1 in macrophage from the ascites of ID8 mice. Data are shown as mean ± SD. Data of (B), (C) and (E) were obtained from three experimental replicates. p values were determined by two-tailed Student’s t test (ns, not significant; **p < 0.01, ***p < 0.001).


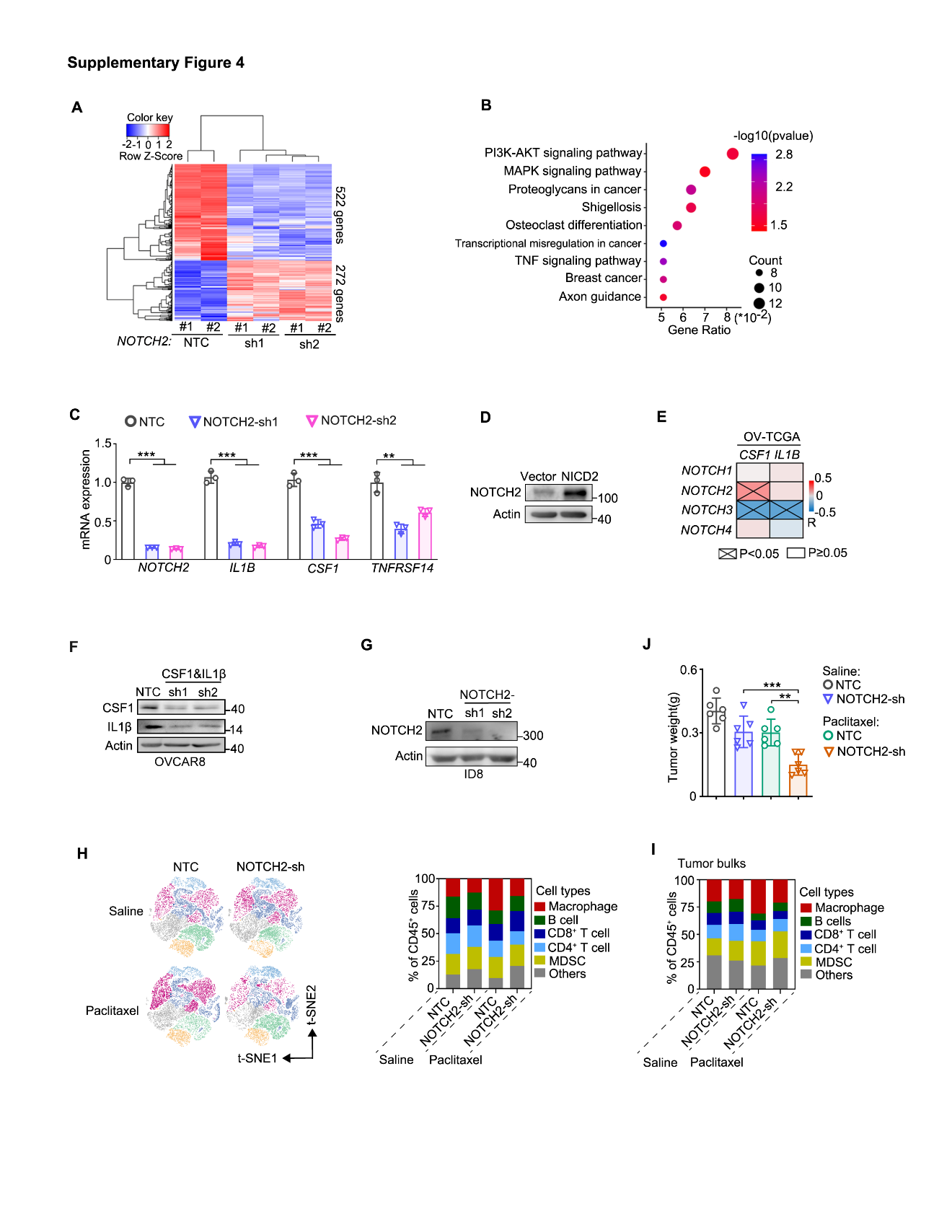


**Figure S4**. **NOTCH2 upregulation activates pro-survival pathways and stimulates the recruitment of pro-tumor macrophages, related to Figure 4. (A)** Heatmap illustrates differentially expressed genes (DEGs) in OVCAR8 cells transfected with NOTCH2-shRNA or non-target control (NTC) vector. **(B)** Kyoto Encyclopedia of Genes and Genomes (KEGG) pathway analysis of DEGs in the RNA-Seq data (NOTCH2 knockdown vs non-target control (NTC)). **(C)** RT–PCR detection of the DEG mRNA levels in OVCAR8 cells after NOTCH2 knockdown. **(D)** OVCAR8 with NOTCH2 knockdown were transfected with a vector or NICD2, and western blot results confirmed the expression of NICD2. **(E)** Pearson correlation between NOTCH receptors and CSF1 and IL1B mRNA expression in ovarian cancer and lung adenocarcinoma of NSCLC from the TCGA database. **(F)** Knockdown level of CSF1 and IL-1B in OVCAR8 cells. **(G)** Knockdown level of NOTCH2 in ID8 cells. **(H)** t-SNE plot showing a low-dimensional representation of the different immune cell types in the ascites of ID8-carrying mice after three weeks of treatment (left panel). The relative percentages of the different immune cell types identified by the flow cytometry analysis of ascites were quantified (right panel). **(I)** Relative percentages of the different immune cell types in the bulk tumor tissues. Percentages of macrophages in the bulk tumor tissues three weeks after treatment, error bars represent mean ± SD. **(J)** Tumor weights from OVCAR8 xenograft mice (n = 6), related to Figure 4J. Data of (C) is shown as mean ± SD, and obtained from three experimental replicates. p values were determined by two-tailed Student’s t test (ns, not significant; **p < 0.01, ***p < 0.001).


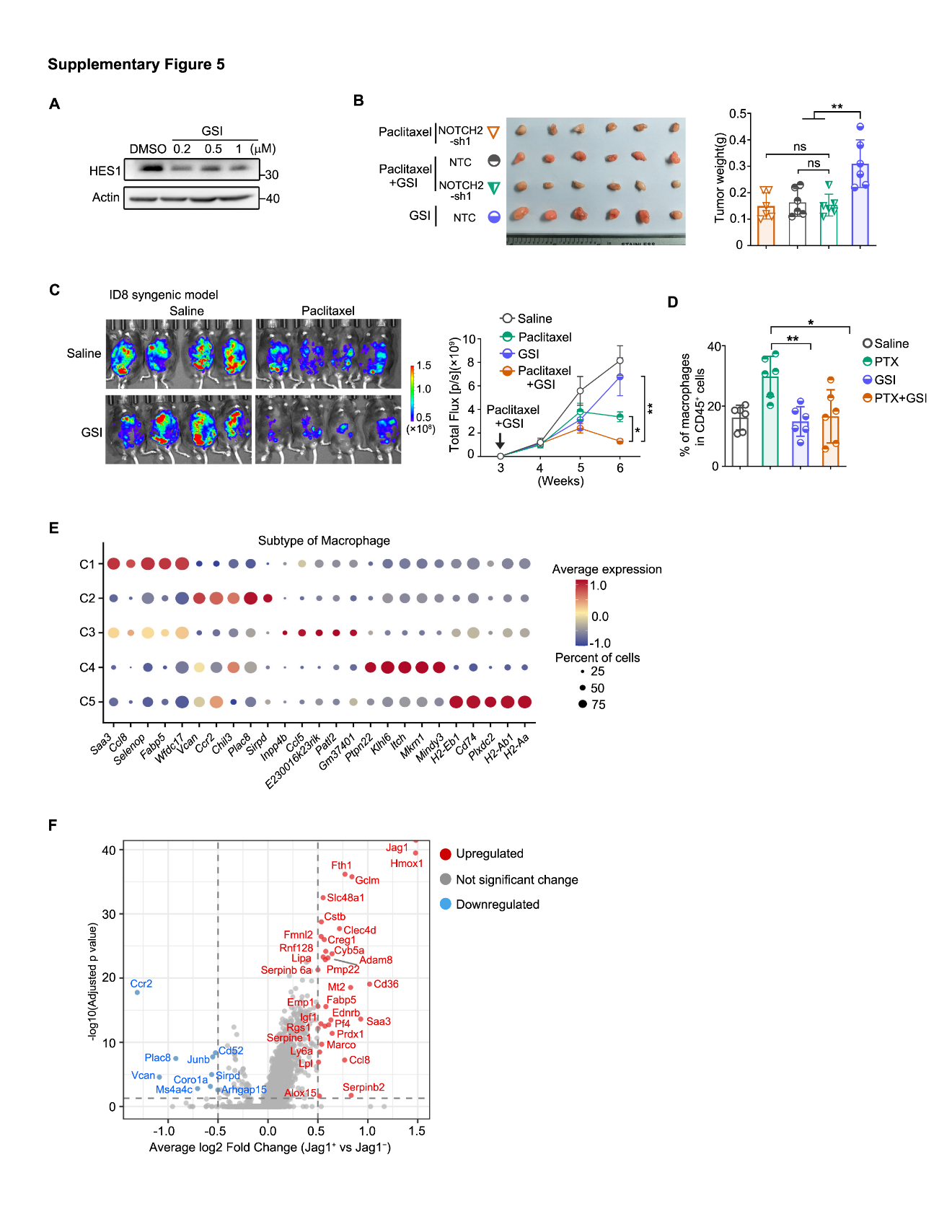
**Figure S5**. **NOTCH2 inhibitor sensitizes tumor response to paclitaxel in xenograft model of ovarian and lung cancer, related to Figure 5. (A)** Western blot analysis of GSI effect on inhibiting NOTCH signaling in OVCAR8 cells. **(B)** Mice carrying OVCAR8 wild-type or NOTCH2-knockout xenografts were treated with GSI and paclitaxel either alone or in combination, n=6 per group, error bars represent mean ± SD. **(C)** Representative images of mice carrying ID8-luciferase cells three weeks after treatment (left panel) and the corresponding tumor growth curves (right panel). n = 6 per group, error bars represent mean ± SEM. **(D)** Percentages of macrophages in the ascites three weeks after treatment were detected using flow cytometry and quantified, error bars represent mean ± SD. **(E)** Dot plot of the marker genes used to annotate the macrophage subsets. Dot size indicates the percentage of cells within each cluster expressing the indicated gene, and the color represents the average expression level. **(F)** Volcano plot showing the transcriptional differences between *Jag1*⁺ and *Jag*1⁻ macrophages. The x-axis represents the average log₂ fold change (*Jag1*⁺ vs. *Jag1*⁻), and the y-axis indicates the –log₁₀ adjusted p value. Significantly upregulated genes in *Jag*1⁺ macrophages are shown in red, downregulated genes in blue, and genes without significant changes in gray. p values were determined by two-tailed Student’s t test (ns, not significant; *p < 0.05, **p < 0.01, ***p < 0.001).


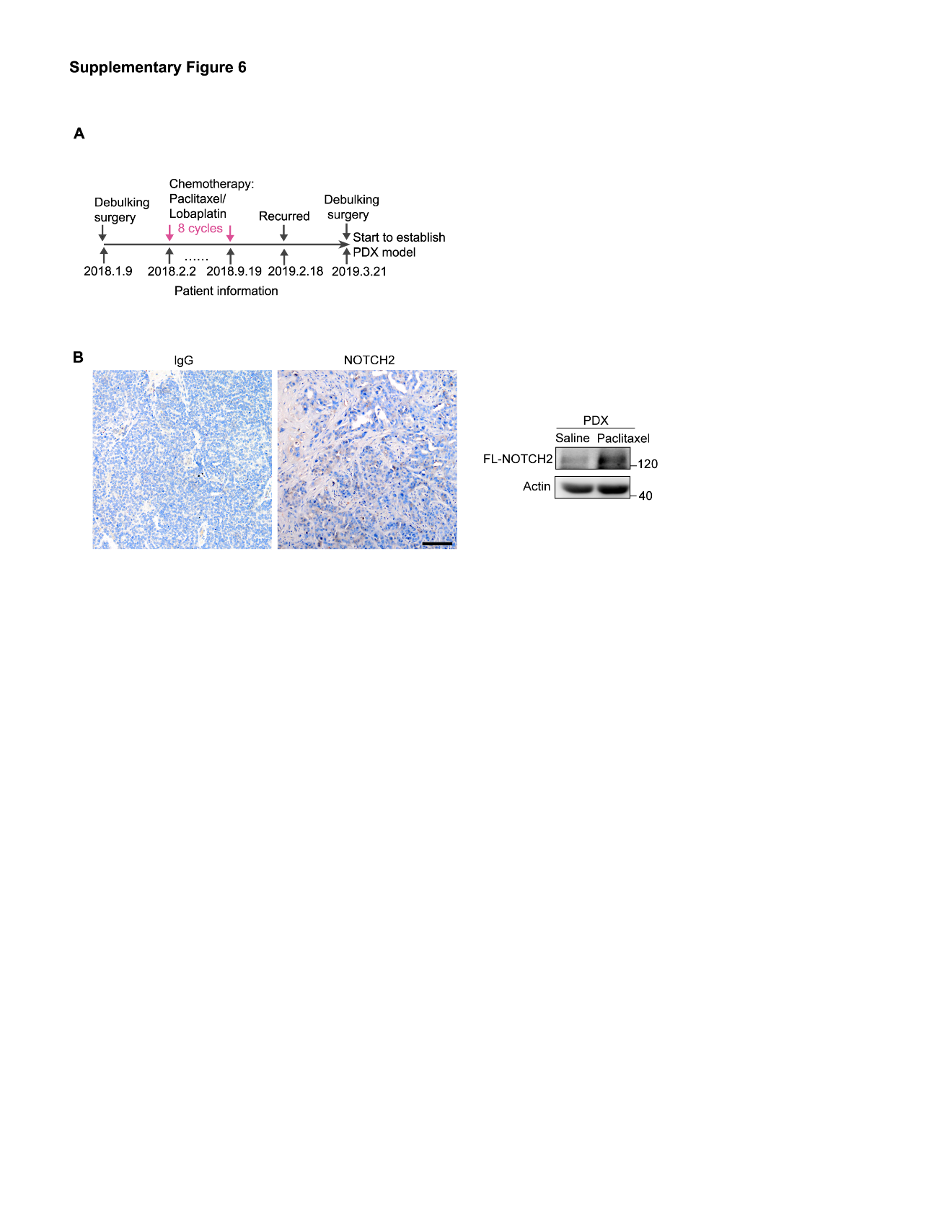


**Figure S6.** **High NOTCH2 level is associated with chemoresistance of ovarian cancer patients, related to Figure 6.**

**(A)** Information of the ovarian cancer patient whose tumor tissue was used to establish the PDX mouse model.

**(B)** Representative immunohistochemical staining of NOTCH2 in corresponding PDX tumors, Scale bar, 100 μm. And Western blot of NOTCH2 in in corresponding PDX tumors.

**Supplemental references**

S1. Park JE, Yi H, Kim Y, Chang H, Kim VN. Regulation of Poly(A) Tail and Translation during the Somatic Cell Cycle. Mol Cell 2016; 62: 462-471.

S2. Tanenbaum ME, Stern-Ginossar N, Weissman JS, Vale RD. Regulation of mRNA translation during mitosis. Elife 2015; 4.
